# Sigma Factor Regulated Cellular Response in a Non-solvent Producing *Clostridium beijerinckii* Degenerated Strain: A Comparative Transcriptome Analysis

**DOI:** 10.3389/fmicb.2017.00023

**Published:** 2017-01-30

**Authors:** Yan Zhang, Shengyin Jiao, Jia Lv, Renjia Du, Xiaoni Yan, Caixia Wan, Ruijuan Zhang, Bei Han

**Affiliations:** ^1^School of Medicine, Institute for Genome Sciences, University of Maryland, BaltimoreMD, USA; ^2^School of Public Health, Health Science Center, Xi’an Jiaotong UniversityXi’an, China; ^3^Department of Bioengineering, University of Missouri, ColumbiaMO, USA

**Keywords:** *Clostridium beijerinckii* NCIMB8052, strain degeneration, transcriptome analysis, microarray, sigma factor

## Abstract

*Clostridium beijerinckii* DG-8052, derived from NCIMB 8052, cannot produce solvent or form spores, a phenomenon known as degeneration. To explore the mechanisms of degeneration at the gene level, transcriptomic profiles of the wild-type 8052 and DG-8052 strains were compared. Expression of 5168 genes comprising 98.6% of the genome was assessed. Interestingly, 548 and 702 genes were significantly up-regulated in the acidogenesis and solventogenesis phases of DG-8052, respectively, and mainly responsible for the phosphotransferase system, sugar metabolic pathways, and chemotaxis; meanwhile, 699 and 797 genes were significantly down-regulated, respectively, and mainly responsible for sporulation, oxidoreduction, and solventogenesis. The functions of some altered genes, including 286 and 333 at the acidogenesis and solventogenesis phases, respectively, remain unknown. Dysregulation of the fermentation machinery was accompanied by lower transcription levels of glycolysis rate-limiting enzymes (*pfk* and *pyk*), and higher transcription of cell chemotaxis genes (*cheA, cheB, cheR, cheW*, and *cheY*), controlled mainly by σ^54^ at acidogenesis. Meanwhile, abnormal spore formation was associated with repressed *spo0A, sigE, sigF, sigG*, and *sigK* which are positively regulated by σ^70^, and correspondingly inhibited expression of CoA-transferase at the solventogenesis phase. These findings indicated that morphological and physiological changes in the degenerated *Clostridium* strain may be related to altered expression of sigma factors, providing valuable targets for strain development of *Clostridium* species.

## Introduction

Solventogenic *Clostridium* species are unique microorganisms due to their natural ability to use a wide range of substrates as carbon sources to produce large amounts of bio-butanol, a process termed acetone-butanol-ethanol (ABE) fermentation (Lee et al., 2008). ABE fermentation is a biphasic process in which solventogenic *Clostridium* species produce acetic and butyric acids intracellularly, and concomitantly release them into the fermentation broth during the exponential growth or acidogenic phase. This is followed by the solventogenic or stationary growth phase, when the acids are re-assimilated into cells and converted to ABE (Ezeji et al., 2010). Butanol, the major ABE fermentation product, is of remarkable interest, since it can be used as alternative fuel due to desirable fuel characteristics and compatibility with gasoline (Bankar et al., 2013). However, solventogenic *Clostridium* species frequently lose their ability to achieve solventogenesis and accumulate excessive amounts of acetic and butyric acids in the fermentation medium after repeated vegetative subculture or during continuous fermentation, a process called strain degeneration (Kashket and Cao, 1995; Sillers et al., 2008).

While degeneration of *Clostridium acetobutylicum* ATCC 824 is caused by the loss of the mega-plasmid pSOL1 that harbors the *sol* operon expressing alcohol/aldehyde dehydrogenase and CoA transferase genes, responsible for acid re-assimilation (Cornillot et al., 1997); degeneration of *C. saccharoperbutylacetonicum* is caused by deficient formation of NADH from pyruvate (Hayashida and Yoshino, 1990). Since *Clostridium beijerinckii* NCIMB 8052 has no mega-plasmid, with solventogenic genes located in the 6.7-Mbp single circular chromosomal DNA (Chen and Blaschek, 1999), this microorganism undergoes degeneration different from *C. acetobutylicum* ATCC 824. Studies applying proteomics and DNA microarrays have been carried out to generate industrially valuable *Clostridia* strains (Tomas et al., 2003; Alsaker and Papoutsakis, 2005; Wang et al., 2012, 2013; Han et al., 2013; Zhang and Ezeji, 2013); however, no genome wide transcriptomic analysis for strain degeneration has been reported.

We recently obtained a degenerated *C. beijerinckii* strain from NCIMB 8052, which only produces 0.58 g/L butanol and 0.87 g/L total ABE at maximum OD_600_ of 2.21. In addition, at the protein level, *C. beijerinckii* 8052 shows lower expression levels of proteins responsible for the disruption of RNA secondary structures, DNA repair, sporulation, signal transduction, transcription regulation, and membrane transport (Lv et al., 2016). Transcriptional profiling of fermentation culture for the degenerated strain may provide more biological evidence and unveil the molecular basis for strain degeneration in this group of microorganisms, especially *C. beijerinckii* NCIMB 8052, whose solventogenic genes are located in the chromosome.

The objective of this study was to explore the molecular basis of degeneration in *C. beijerinckii* NCIMB 8052 by applying genome-wide transcriptional analysis of the WT-8052 and its degenerated strain DG-8052. Comparison of transcriptome profiles associated with ABE production would provide valuable insights regarding potential targets for metabolic engineering of *C. beijerinckii* NCIMB 8052, to prevent strain degeneration and develop robust industrial butanol producing bacteria.

## Materials and Methods

### Bacterial Strains and Culture Conditions

*Clostridium beijerinckii* NCIMB 8052 and its degenerate strain *C. beijerinckii* DG-8052 were used in this study. *C. beijerinckii* DG-8052 is a non-ABE producing strain, and was generated as described previously (Lv et al., 2016). Tryptone–Glucose–Yeast Extract medium was used to culture *C. beijerinckii* NCIMB 8052 and DG-8052 cells, in an anaerobic chamber.

### Batch Fermentation

To perform transcriptional analyzes of *C. beijerinckii* WT-8052 and DG-8052, 6% (v/v), actively growing pre-cultures were sub-cultured into the P2 fermentation medium; unless otherwise stated, all experiments were carried out in triplicate, and temperature was maintained at 35 ± 1°C without shaking or pH control. The pH profile of cultures was monitored on a Beckman Φ500 pH meter. Growth of *C. beijerinckii* strains was estimated at OD_600_ on a F-7000 spectrophotometer (Ezeji et al., 2004; Han et al., 2011).

### Total RNA Isolation and Purification

After 12 and 24 h of fermentation, 10 mL of *C. beijerinckii* (WT-8052 and DG-8052) culture were centrifuged at 5000 g and 4°C for 10 min. The resulting cell pellets were kept for total cellular RNA extraction with RiboPure^TM^ bacteria RNA Purification Kit (Ambion^®^, Life Technologies, Inc., USA) according to the manufacturer’s instructions. RNA concentration was measured on a NanoDrop 1000 (NanoDrop Technologies, Wilmington, DE, USA). RNA quality was assessed by 1.2% denatured formaldehyde gel electrophoresis. RNA samples for microarrays had 23S:16S rRNA ≥ 2:1, and A260:A280 ≥ 1.80.

### Comparative Microarray Hybridization

Complementary DNA (cDNA) synthesis and amino-allyl labeling were performed as described previously (Almeida et al., 2006). Using the crystal Core^®^ cDNA amplified RNA labeling kit (CapitalBio, Beijing, China), 1 μg of total RNA was reverse transcribed into cDNA and labeled with 100 μM each dATP, dTTP, and dGTP, and 25 μM Cy3- or Cy5-labeled dCTP. For two-color microarray hybridization, Cy5 labeled DG-8052 cDNA and Cy3 labeled WT-8052 cDNAs from samples collected at both acidogenic (12 h, three samples) and solventogenic (24 h, three samples) growth phases were used. Microarray probes were designed with the Agilent eArray software^1^, based on the genomic sequence of *C. beijerinckii* NCIMB 8052. Hybridization was performed by CapitalBio corporation (Beijing, China) and Agilent Technologies (Beijing, China) using a custom-made Agilent chip (15000 probes/array). To reduce technical variations, three identical replicates of each *C. beijerinckii* probe (60mer) with 60 negative and positive control probes were included in each array. The 12 and 24 h triplicate samples were hybridized to six arrays; the hybridized slides were scanned on an Agilent G2565CA Microarray Scanner, and the GenePix^®^ Pro 7 Microarray Acquisition and Analysis Software (Molecular Devices, Sunnyvale, CA, USA) was used for data extraction.

### Microarray Data Analysis

The extracted images were analyzed with Molecular Annotation System V4.0 (CapitalBio, Beijing, China). Since normalization of signal intensities is needed to render gene expression levels measured by two different dyes comparable, raw signal intensities of messenger RNAs (genes) were normalized with LOWESS (Locally Weighted Scatterplot Smoothing) as described previously (Berger et al., 2004). For each array, gene expression ratios were calculated by dividing Cy5 intensities (DG-8052 signals) by those of Cy3 (WT-8052 signals). The expression ratio for each gene was the average expression ratio of the three replicates. To identify differentially expressed (up- and down-regulated) genes and facilitate a fair comparison between WT-8052 and DG-8052 genes, fold changes were calculated as previously proposed; a cut-off of 2 was set to define differential expression (Quackenbush, 2002). Gene expression profiles of DG-8052 and WT-8052 were analyzed by pairwise and point-by-point comparisons using SAM (Significant Analysis of Microarrays) version 2.23b. To reduce false positives, *p*-value was adjusted to *q*-value. Therefore, genes with greater than twofold change and *q*-value < 0.05 were considered to be significantly regulated. Gene Ontology enrichment and Kyoto Encyclopedia of Genes and Genomes (KEGG) analyses were performed to elucidate the functions of differentially expressed messenger RNAs (molecular function). Using DAVID Functional Annotation Bioinformatics Microarray Analysis, further functional enrichment analyses were performed to identify cellular and metabolic pathways, especially the statistically overrepresented groups (Zhang and Ezeji, 2013).

### Microarray Data Accession Number

All protocols associated with the development of this microarray platform, including but not limited to information regarding probe sequences and synthesis, labeling, hybridization and scan protocols, as well as microarray data have been submitted to NCBI’s Gene Expression Omnibus database at http://www.ncbi.nlm.nih.gov/geo/ (GEO accession number GSE63671).

### Quantitative Real-Time PCR (qRT-PCR)

To validate microarray data, 10 up-regulated (Cbei_4123, Cbei_0411, Cbei_0311, Cbei_3120, Cbei_0441, Cbei_0331, Cbei_4824, Cbei_2740, Cbei_4871, and Cbei_3356) and 10 down-regulated (Cbei_3835, Cbei_1930, Cbei_0677, Cbei_2826, Cbei_2600, Cbei_1583, Cbei_1079, Cbei_0284, Cbei_3832, and Cbei_2261) genes were randomly selected for qRT-PCR. *C. beijerinckii* (WT-8052 and DG-8052) cultures were grown anaerobically, and cells were harvested after 12 and 24 h of fermentation as described above. RNA was isolated and quantitated as described above. For qRT-PCR, primers specific to the 20 selected genes were designed (Supplementary Table S1). The 16S rRNA gene of *C. beijerinckii* 8052 was used as internal control. Specifically, total RNA (2 μg) was reverse transcribed into cDNA with the SuperScript^TM^ III reverse transcriptase (Invitrogen Corporation, Carlsbad, CA, USA). The expression levels of all tested genes were normalized to the amounts of the internal control gene 16S rRNA (Han et al., 2013).

### Cellular Aging and Scavenging Ability of Reactive Oxygen Species

Given that total oxygen species (ROS) are continually generated and possibly degraded under normal physiological conditions, and considering their roles in cell signaling, homeostasis and aging (Kohanski et al., 2008), ROS (^.^OH), generated by100 μM of ferrous perchlorate (II) and 1 mM of H_2_O_2_ in *C. beijerinckii* DG-8052 and WT-8052 during ABE fermentation were assessed. Briefly, 12 and 24 h *C. beijerinckii* DG-8052 (100 ml) and WT-8052 cultures (100 ml) grown in P2 medium were used for analyses. About 1 ml culture was centrifuged at 5000 g for 5 min, with the resulting cell pellet suspended in 500 μl of 1 × PBS buffer (pH 7.2) followed by addition of 100 μM ferrous perchlorate (II) (50 μl) and 1 mM H_2_O_2_ (38 μl) to generate hydroxyl radical. About 1 μl of the cell-permeable fluorogenic probe aminophenyl fluorescein (APF, Molecular Probes^TM^, Invitrogen, Life Technology; 10 μM), a fluorescent reporter dye, was added to the mixture for 30 min at 35°C. Then, the cells were washed three times with P2 medium to remove excess APF probe. Fluorescence (intracellular ROS levels) was measured at excitation/emission wavelengths of 490/515 nm on a microplate reader (Synergy HT, BioTek, USA) (Liu et al., 2013). The produced .OH were toxic to *C. beijerinckii* cells. In *C. acetobutylicum*, cells could detoxify by specific pathway (Riebe et al., 2009). Higher levels of fluorescence indicated lower scavenging ability of ROS in *C. beijerinckii* DG-8052 and WT-8052 cells. And this experiment could investigate the differences in cell aging between DG-8052 and WT-8052.

### Statistical Analysis

Data were analyzed by ANOVA, with *p* < 0.05 considered statistically significant. Unless otherwise stated, all data were expressed as mean ± SD (*n* ≥ 3). All analyses were performed using the General Linear Model (GLM) procedure of SAS Version 9.1.3 (SAS Institute Inc., Cary, NC, USA).

## Results

### Growth of Degenerated Strain *C. beijerinckii* DG-8052

**FIGURE 1:**
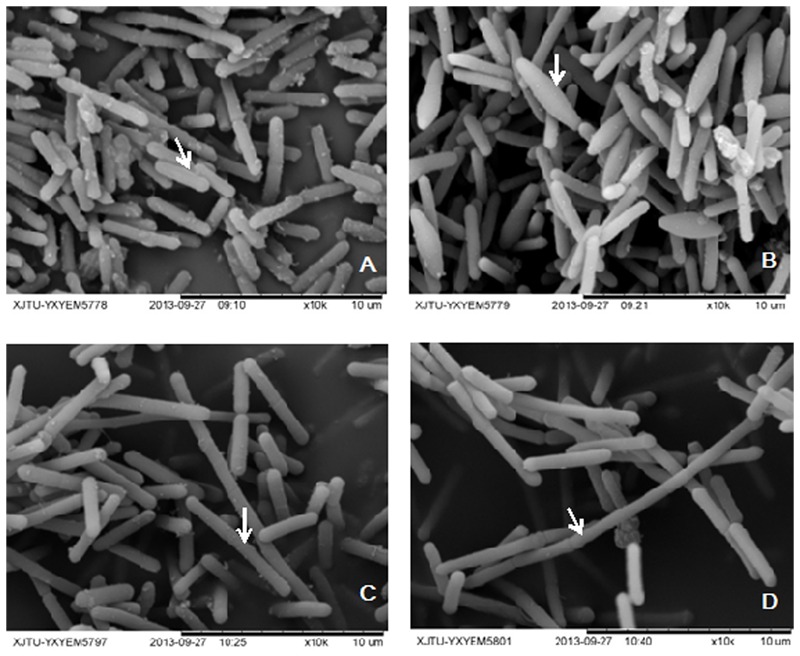
**Electron micrograph of *Clostridium beijerinckii* NCIMB 8052 WT-8052 and DG-8052 cells.** WT-8052 cells were taken at 12 h **(A)** and 24 h **(B)**; DG-8052 cells were taken at 12 h **(C)** and 24 h **(D)**. WT-8052 cells at 12 h were in rod-shape, and in rod-shape with a rounded end at 24 h, which indicating the developing of pre-endospores; DG-8052 cells were all in non-split and elongated rod-shape both at 12 and 24 h, which were disable to produce solvents and form spores. The typical cells were indicated by white arrows.

**FIGURE 2:**
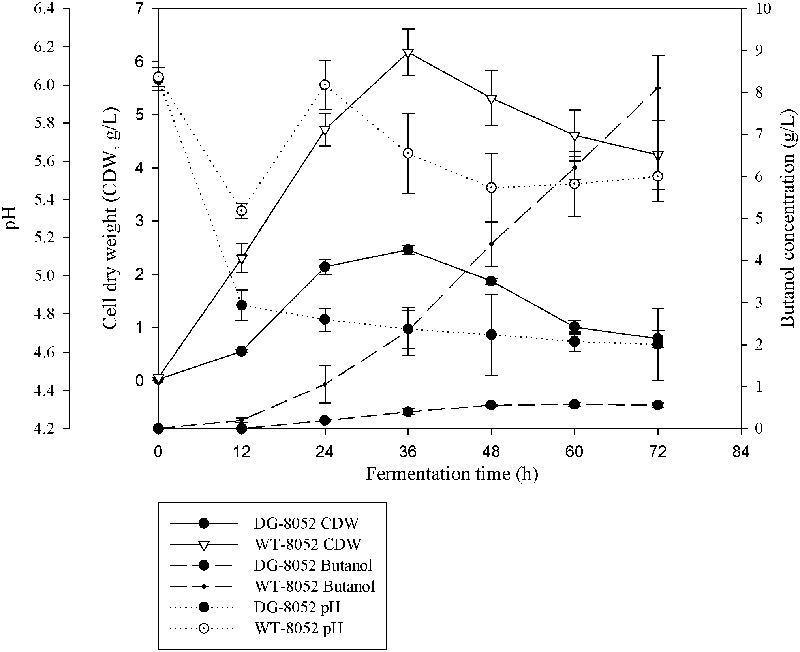
**Fermentation profiles for *C. beijerinckii* NCIMB 8052 and its degenerate stain DG-8052 in P2 medium**.

In solventogenic *Clostridium* species, the spore development (sporulation) usually accompanies with the production of solvents. Electron microscopy showed the typical vegetative cells of WT-8052 and DG-8052, and were rod shaped at 12 h (8–10 μm × 0.8 μm, **Figures 1A,C**). At 24 h, WT-8052 cells were in the stationary phase and started to produce solvents, and were rod-shaped with a round end (**Figure 1B**), indicating the developing of pre-endospores. While DG-8052 showed a large proportion of non-split and elongated rod cells (8–12 μm × 0.8 μm, **Figure 1D**). Fermentation results also verified that DG-8052 lost the solvent producing ability, with reduced cell optical density (**Figure 2**).

### Overall Gene Transcription Dynamics

**FIGURE 3:**
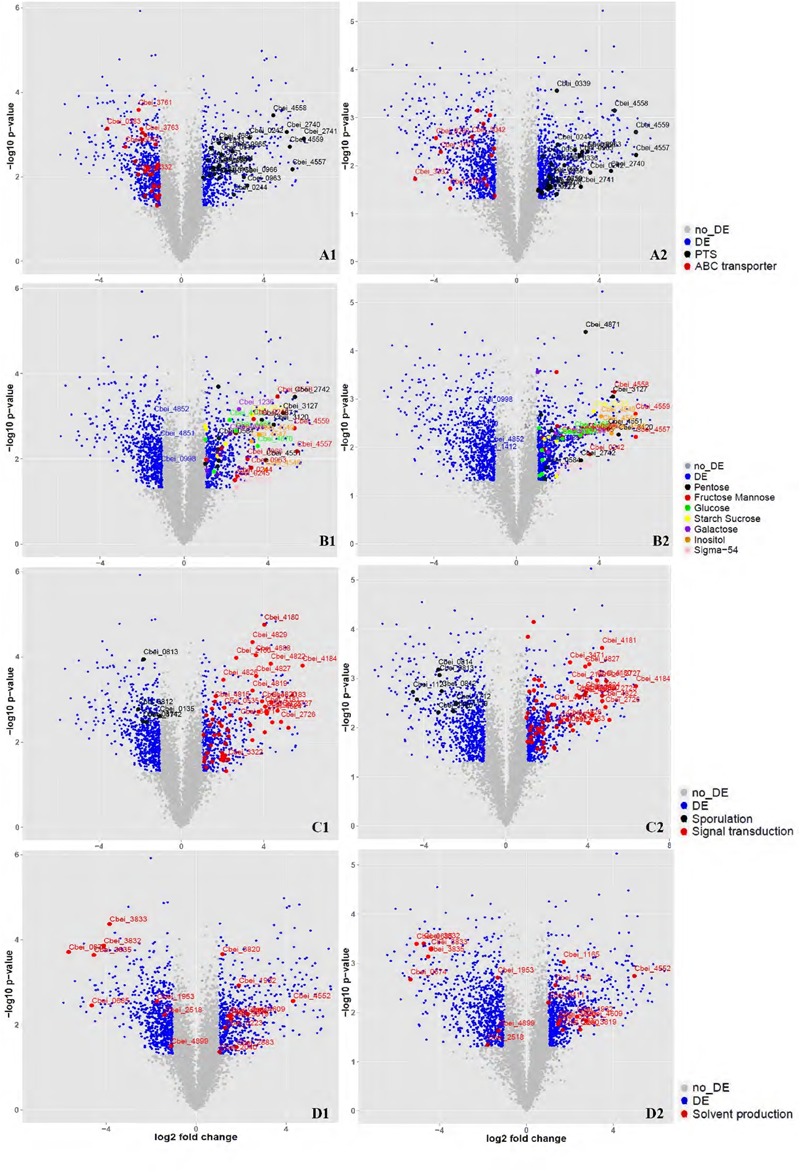
**Overview of the related genes expressed significantly at DG-8052 compared with WT-8052.** Cell samples from both strains were taken at 12 and 24 h to compare transcriptome profiles at acidogenic and solventogenic phases, respectively. Genes with significantly differential expression (fold change > 2 and *p*-value < 0.05) between the two strains were shown in blue while the others with non-significantly differential expression were shown in gray. Sugar transporter (12 h, **A1**; 24 h, **A2**), Sigma-54 and sugar metabolism (12 h, **B1**; 24 h, **B2**), Sporulation and signal transduction (12 h, **C1**; 24 h, **C2**), Solvent production (12 h, **D1**; 24 h, **D2**). DE, differential expression; no_DE, no differential expression; PTS: phosphotransferase system; ABC transporter: ATP-binding cassette transporters; Pentose: pentose phosphate pathway; Fructose Mannose: fructose and mannose metabolism; Glucose: glycolysis/gluconeogenesis; Starch sucrose: starch and sucrose metabolism; Galactose: galactose metabolism; Inositol: inositol phosphate metabolism; Signal transduction: including chemotaxis, two-component signal transduction system and sensor proteins.

**FIGURE 4:**
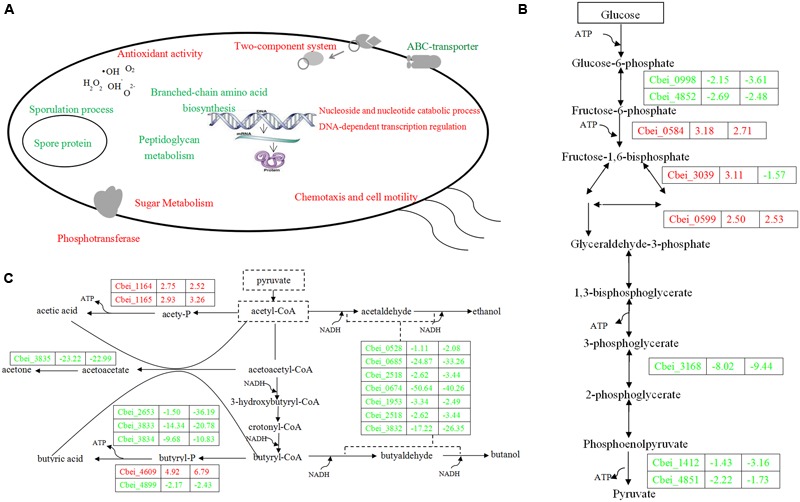
**Sketch map of *C. beijerinckii* NCIMB DG-8052 cell metabolic processes (A)**, glycolysis pathway **(B)** and solventogenic pathway **(C)**. Blue color indicates the down-regulated function/gene; red color indicates the up-regulated function/gene.

Total RNA was isolated from DG-8052 and WT-8052 cell specimens, at 12 and 24 h, separately. Then, transcriptome profiles of WT-8052 and DG-8052 were assessed by microarrays. This study examined the expression of 5168 genes representing 98.6% of the *C. beijerinckii* NCIMB 8052 genome (Supplementary Tables S3 and S4). DG-8052 showed 548 and 702 genes significantly up-regulated in the acidogenic and solventogenic phases, respectively. According to pathway enrichment and Gene Ontology terms, these genes were significantly overrepresented in cellular functions such as sugar transport system, sugar metabolic pathways, and bacterial chemotaxis. Meanwhile, there were 699 and 797 genes significantly down-regulated in the acidogenic and solventogenic phases, respectively. The repressed genes were mainly enriched in functions, such as sporulation-related biosynthesis, oxidoreduction, and solvent production (**Figures 3** and **4**; **Table 1**). Not all the regulated genes had known function, and COG analysis revealed that 286 and 333 regulated genes in the acidogenesis and solventogenesis phases, respectively, had unspecified functions.

**Table 1:** Kyoto Encyclopedia of Genes and Genomes (KEGG) pathway showing significant changes in *Clostridium beijerinckii* DG-8052.

Culture	KEGG ID	Pathway definition	*p*-value	Benjamini^∗^	Count^∗∗^
*Up-regulated*				
12 h	cbe02030	Bacterial chemotaxis	2.0E-4	1.0E-1	19
	cbe00051	Fructose and mannose metabolism	4.0E-4	1.1E-2	17
	cbe02060	Phosphotransferase system (PTS)	5.3E-4	9.3E-3	24
	cbe00040	Pentose and glucuronate interconversions	8.0E-4	1.0E-2	12
	cbe00562	Starch and sucrose metabolism	3.1E-2	2.4E-1	10
	cbe00030	Pentose phosphate pathway	3.8E-2	2.6E-1	11
	cbe00052	Galactose metabolism	4.3E-2	2.5E-1	8
24 h	cbe02030	Bacterial chemotaxis	1.3E-5	7.7E-4	24
	cbe02060	PTS	2.1E-4	6.2E-3	29
	cbe00051	Fructose and mannose metabolism	6.9E-4	1.4E-2	19
	cbe00040	Pentose and glucuronate interconversions	1.6E-3	2.4E-2	13
	cbe00562	Starch and sucrose metabolism	7.3E-3	8.4E-2	13
	cbe00030	Pentose phosphate pathway	1.4E-2	1.1E-1	14
	cbe00052	Galactose metabolism	1.8E-2	1.3E-1	10
	cbe02020	Two-component system	3.8E-2	2.3E-1	17
	cbe00010	Glycolysis/gluconeogenesis	5.5E-2	2.9E-1	13
	cbe00520	Amino sugar and nucleotide sugar metabolism	5.6E-2	2.7E-1	15
*Down-regulated*				
12 h	cbe02010	ABC transporters	3.8E-5	2.1E-3	32
	cbe00290	Valine, leucine, and isoleucine biosynthesis	8.6E-4	2.3E-2	9
	cbe00240	Pyrimidine metabolism	7.8E-2	7.7E-1	10
24 h	cbe00633	Trinitrotoluene degradation	4.2E-4	2.2E-2	5
	cbe00680	Methane metabolism	8.2E-3	2.0E-1	4
	cbe00290	Valine, leucine, and isoleucine biosynthesis	1.3E-2	2.1E-1	7
	cbe00340	Pyrimidine metabolism	2.1E-2	2.4E-1	11
	cbe00051	Fructose and mannose metabolism	2.3E-2	2.2E-1	11
	cbe00260	Glycine, serine, and threonine metabolism	4.9E-2	3.6E-1	6
	cbe00230	Purine metabolism	5.3E-2	3.4E-1	11

*^∗^Significant groups were selected based upon Benjamini (<0.05). ^∗∗^Number of genes within the given KEGG ID showing a significant change in their expression level.*

### Sugar Phosphotransferase System (PTS) and Sugar Metabolism Related Genes

At the vegetative growth stage of DG-8052, expression of phosphotransferase system (PTS) operons was significantly elevated. The main sugar families affected by altered PTS expression were mannose/fructose/sorbose (Man), lactose/cellobiose (Lac), mannitol (Fru), sucrose/glucoside (Glc), and glucitol/sorbitol (Gut) families (**Figure 2**; Supplementary Table S5a). The sigma-54 dependent PTS operon of the Lac family was characterized in *Listeria monocytogenes*; it regulates the *lpo* operon associated with a cognate activator LacR and positive regulated the sugar transporter (Dalet et al., 2003). In DG-8052, sigma 54 (Cbei_0595) showed an increased expression with 5.75 fold change at 12 h. Four sigma-54 dependent PTS lactose operons (Cbei_0220-0222, Cbei_0950-0953, Cbei_4684-4685, and Cbei_4639-4640) had significantly higher expression levels in DG-8052 (**Figure 3A**). In the Man family operons (Cbei_0953-0959, Cbei_0962-0967, and Cbei_4555-4560), the sigma 54 factor interaction domains (Cbei_0953, 0954, 0962, 4555, 3875, and 4915) were all up-regulated with fold changes of +2.68, +2.38, +2.17, +6.36, +2.64, +2.87, respectively (**Figure 3A**). Beside Sigma-54 factor, several operons were found with elevated expression levels in other families positively regulated by the transcriptional anti-terminator *bglG* (**Figure 3**). Such operons include Fru family Cbei_0239–0245 and Cbei_2739-2741, Gut (Glucitol) family Cbei_0333–0340, and Glc family Cbei_2833. The BglG near the operons showed high expression level, including Cbei_0242 (+6.34), Cbei_2738 (+19.95), Cbei_0334 (+2.63), Cbei_2834 (+2.67).

Beside sugar transporter genes, genes related to sugar metabolic pathways were up-regulated as well in DG-8052, including glycolysis, pentose phosphate pathway, starch and sucrose metabolic pathway, galactose metabolic pathway, and inositol phosphate metabolic pathway (**Figure 3B**; Supplementary Table S5b). In DG-8052, the glycolysis rate-limiting enzymes *pfk* (Cbei_0998, -2.15, -3.61; Cbei_4852, -2.69, -2.48), *pyk* (Cbei_4851, -2.22, -1.73; Cbei_1412, -1.43, -3.16) and *pgam* (Cbei_3168, -8.02, -9.44) were all down-regulated (**Figures 3B** and **4B**). Lower transcription of rate-limiting enzymes in the glycolytic pathway – *pfk* and *pyk* may cause decreased expression of phosphofructokinase and pyruvate kinase, yielding less ATP during glycolysis. This may explain the poor growth and slow metabolism using carbon source in DG-8052 cells. SigE (Cbei_1120, 1.99, -9.81) was down-regulated significantly at 24 h. There may be a similar positive regulation by SigE in degenerated clostridia.

### Sigma Factors and Sporulation Genes

In this study, transcription levels of sporulation factors were reduced in the solventogenesis phase, such as YlmC/YmxH (Cbei_1122, -28.65), YunB (Cbei_4229, -4.51), and YtfJ (Cbei_1877, -2.98); sporulation stage II, protein E (Cbei_0097, -21.02), protein R (Cbei_0395, -2.51), protein D (Cbei_0422, -20.31), and protein P (Cbei_0832, -11.68); stage III, protein AA (Cbei_1692, 21.72), protein SpoAB (Cbei_1693, 12.98), protein AC (Cbei_1694, 4.40), protein AD (Cbei_1695, 26.32), protein AE (Cbei_1696, 16.05), protein AG (Cbei_1697, 13.58), protein AH (Cbei_1698, 27.17), and transcription regulator SpoIIID (Cbei_0424, -33.32); stage IV, protein A(Cbei_1136, -8.57) and protein B(Cbei_1711, -6.00); stage V sporulation, protein E (Cbei_1583, -2.21). Sporulation is generally regulated by the transcription factor Spo0A and other sigma factors, such as SigH, SigF, SigE, and SigG, in gram-positive bacteria (Li and McClane, 2010; Kirk et al., 2014). Compared with WT-8052, DG-8052 had the decreased transcription of SigH (sigma 70, Cbei_0135, -2.06, -1.79), Spo0A (Cbei_1712, -3.16, -5.35), SigE (Cbei_1120, 1.99, -9.81), and SigG (Cbei_1121, -1.02, -23.22) both in the acidogenic and solventogenic phases (**Figure 3C**; Supplementary Table S5c).

### Chemotaxis

This study showed that bacterial chemotaxis was one of the most enriched pathways (*p*-value < 0.01) up-regulated in DG-8052 at both 12 and 24 h. There were 19 chemotaxis genes up-regulated at 12 h, and induced at 24 h including all the 19 above genes, which encompassed genes encoding methyl-accepting chemotaxis sensory transducer (MCP), auto-phosphorylable sensory histidine kinase CheA, adaptor protein CheW, methyltransferase CheR, methylesterase CheB, and response regulator CheY, with up-regulation fold changes from +2.01 to +82.01 (**Figure 3C**; Supplementary Table S5d).

### Solvent Producing Pathway

During acidogenesis, genes encoding acid producing enzymes showed higher expression levels in DG-8052 than in WT-8052, e.g., acetate kinase (Cbei_1165, +2.93), phospho-trans-acetylase (Cbei_1164, +2.75), and butyrate kinase (Cbei_4609, +4.92) (**Figure 3D**; Supplementary Table S5). When acids accumulate to a certain level, CoA-transferase begins to re-assimilate them into cells (Millat et al., 2014). In *C. beijerinckii* 8052, a *sol* operon organized in order of *ald* (aldehyde dehydrogenase, Cbei_3832)-*ctfA* (CoA-transferase subunit A, Cbei_3833)-*ctfB* (CoA-transferase subunit B, Cbei_3834); In DG-8052, the coordinated expression was observed for the *sol* operon genes, Cbei_3832-Cbei_3833-Cbei_3834) were all down-regulated at fold changes from -17.22, -14.34, -9.68 (acidogenesis) to -4.91, -20.78, -10.83 (solventogenesis), respectively. In DG-8052, there had several *aldA* copes (aldehyde dehydrogenase, Cbei_1953, Cbei_3832, Cbei_0674, and Cbei_2518), which probes were all down-regulated, especially Cbei_0674 (-50.64, -40.26) (Supplementary Table S5f). In addition, acetoacetate decarboxylase (Cbei_3835, -23.22, -22.99) was greatly down-regulated as well. While there had no obvious changes between DG-8052 and WT-8052 in enzymes, which including acetyl-CoA acetyltransferase (Cbei_0411, Cbei_3630) catalyzing acetyl-CoA into acetoacetyl-CoA; 3-hydroxybutyryl-CoA dehydrogenase (Cbei_0324, Cbei_0325, Cbei_2037) catalyzing acetoacetyl-CoA into 3-hydroxybutyryl-CoA; Enoyl-CoA hydratase (Cbei_2231, Cbei_2230) catalyzing 3-hydroxybutyryl-CoA into crotonyl-CoA; butyryl-CoA dehydrogenase (Cbei_0322, Cbei_2035, Cbei_2883) catalyzing crotonyl-CoA into butyryl-CoA (**Figures 3D** and **4C**; Supplementary Table S5f).

### Cellular Aging and Scavenging Ability of Reactive Oxygen Species

**FIGURE 5:**
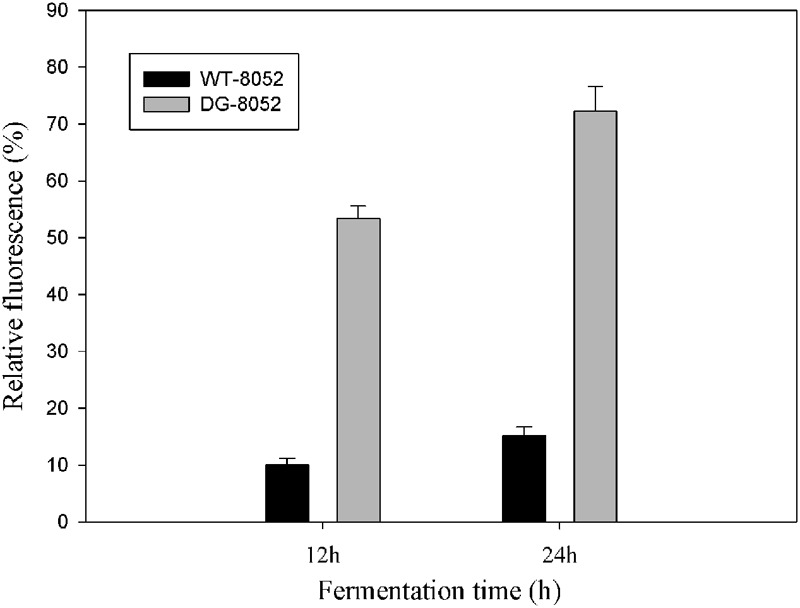
**Comparison of the reactive oxygen species produced and accumulated in DG-8052 and WT-8052.** 100 μM of ferrous perchlorate (II) and 1 mM of H_2_O_2_ was used to generate hydroxyl radical, and it was mixed with aminophenyl fluorescein (APF). The green fluorescence was used as positive control. The fluorescence signal of mixture (WT-8052 and DG-8052 cells culture in 12 and 24 h with APF separately) were compared with positive control to get the relative fluorescence (%).

To investigate the differences in cell aging between DG-8052 and WT-8052, the concentration of total reactive oxygen species (ROS) were measured. ⋅OH was generated by100 μM of ferrous perchlorate (II) and 1 mM of H_2_O_2_, and the produced ⋅OH were toxic to *C. beijerinckii* cells. *C. beijerinckii* could detoxify by the similar pathway (NADH oxidase). In DG-8052, a higher fluorescence signal was detected indicating higher amount of ROS residues than that of DG-8052 (**Figure 5**). It was also observed at the same fermentation time, DG-8052 cells had less viability than WT-8052 cells, which may result from the high cellular oxidative stress in DG-8052.

### Validation of Gene Expression Data from Microarray Analysis by Q-RT-PCR

**FIGURE 6:**
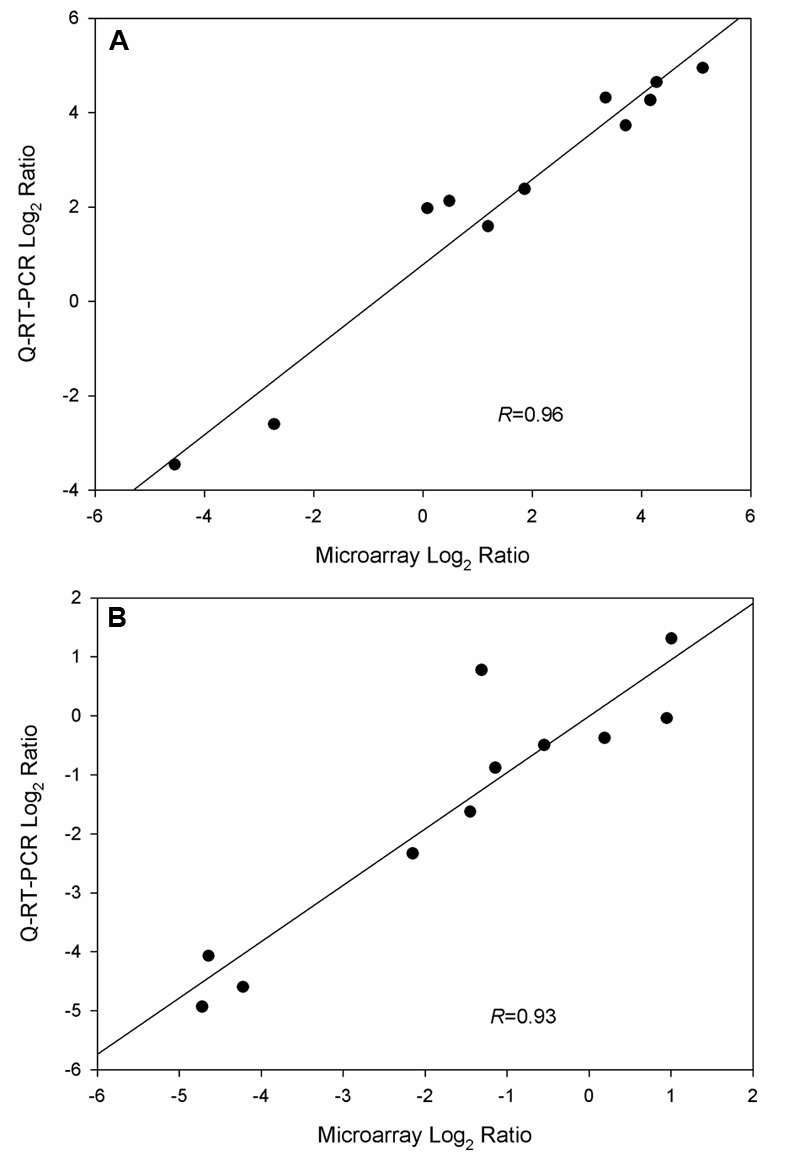
**Validation of microarray data from *C. beijerinckii* DG-8052 at two growth stages using Q-RT-PCR. (A)** Acidogenic phase and **(B)** solventogenic phase.

To validate microarray data, Q-RT-PCR was applied to quantify gene expression levels in triplicate cultures of WT-8052 and DG-8052 in the same conditions used for the microarray analysis. A total of 14 and six genes were selected in the acidogenic (12 h) and solventogenic (24 h) phases, respectively for evaluation. The results from both expression assays, microarrays and Q-RT-PCR, showed that gene expression levels in WT-8052 vs DG-8052 had a high correlation at both 12 h (*R* = 0.96) and 24 h (*R* = 0.93) (**Figure 6**, Supplementary Figure S1; Supplementary Table S1).

## Discussion

This study showed a dysfunction in spore formation and solvent production in DG-8052, which was similar to degenerated *C. acetobutylicum* M5, a strain with loss of the mega-plasmid pSOL1 containing the key solvent formation genes under the *sol* operon (*aad-ctfA-ctfB*) and *adc* gene (Sillers et al., 2008), as well as degenerated *C. acetobutylicum* SKO1, a strain with inactivated *spo0A* gene (Tomas et al., 2003). In *C. beijerinckii* NCIMB 8052, however, no mega-plasmid has ever been found, and therefore its degeneration could not be ascribed to the loss of key genes on the mega-plasmid, leaving the mechanism largely unclear.

Compared with WT-8052, genome resequencing of DG-8052 (SRP082285) showed no general regulator mutated. As we reported recently (Jiao et al., 2016), a total of 20 SNPs (17 non-synonymous, two premature stop, and one intergenic) and 16 InDels (10 coding and six non-coding) were found in the DG-8052 genome; among them, eight SNPs were effected by strain degeneration (Supplementary Table S2), and no transcription change was observed in the remaining 12 DG-8052 mutations. The transcriptional analysis suggested there’s little doubt that *C. beijerinckii* 8052 degeneration drastically altered gene regulation, resulting in corresponding morphological and physiological changes. At the proteomic level, 3% of proteins were shown to be differentially expressed in DG-8052, resulting in the corresponding morphological and physiological changes (Lv et al., 2016). At the transcriptomic level, further mining of the current data will help reveal the regulatory mechanisms and sensitive gene targets for developing degenerated *Clostridium* strains.

Transcriptional analysis of degenerated *C. beijerinckii* strain DG-8052 suggests morphological and physiological changes were related to the disturbed expression of sigma factors, from aspects of sugar transport and metabolism, sporulation, chemotaxis, and solventogenic pathway.

The sigma-54 dependent PTS operon of the Lac family was characterized in *Listeria monocytogenes*; it regulates the *lpo* operon associated with a cognate activator LacR and positive regulated the sugar transporter (Dalet et al., 2003). In our study, four sigma-54 dependent PTS lactose operons, three sigma-54 dependent Man family operons were all up-regulated in DG-8052. Beside Sigma-54 factor, several operons were found with elevated expression levels in other families positively regulated by the transcriptional anti-terminator *bglG*. These findings suggested that PTS operons for sugar families may be regulated by sigma-54 and/or *bglG*, which deserves further experimental verification.

Beside sugar transporter genes, genes related to sugar metabolic pathways were up-regulated as well in DG-8052, including glycolysis, pentose phosphate pathway, starch and sucrose metabolic pathway, galactose metabolic pathway, and inositol phosphate metabolic pathway. Lower transcription of rate-limiting enzymes in the glycolytic pathway – *pfk* and *pyk* may cause decreased expression of phosphofructokinase and pyruvate kinase, yielding less ATP during glycolysis. This may explain the poor growth and slow metabolism using carbon source in DG-8052 cells. In *Cyanobacterium synechocystis* sp. PCC 6803, sugar catabolic pathways were positively regulated by SigE. Indeed, *sigE* mutation in *C. synechocystis* causes reduced transcription of these genes, including *pfkA, gap, pyk, zwf, opcA, gnd, tal, glgX*, and *glgp* (Osanai et al., 2005). In DG-8052, the down-regulated SigE may work as a similar positive regulator.

Sporulation is generally regulated by the transcription factor Spo0A and other sigma factors, such as SigH, SigF, SigE, and SigG, in gram-positive bacteria (Li and McClane, 2010; Kirk et al., 2014). *Spo0A* gene inactivation resulted in the degenerated *C. acetobutylicum* SKO1 strain, which shows an asporogenous, filamentous and largely deficient solventogenic phenotype (Tomas et al., 2003). In *B. subtilis*, the sigma factor SigH (*spo0H*) regulates *spo0A* (Saujet et al., 2011); SigH and Spo0A regulate SigE, which in turn induces the transcription of SigK; SigF controls SigG; SigH and Spo0A may also bind to the promoter region of the *sigF* operon and positively regulate the anti-anti-sigma factors SpoIIAA, SpoIIAB, and SigF (Sierro et al., 2008). In DG-8052, the repressed SigH and Spo0Adecreased the transcription levels of SigE, SpoIIAA, SpoIIAB and SigF, both in the acidogenic and solventogenic phases; meanwhile, down-regulated SigF positively regulated SigG. SpoIIGA is required for intermembrane proteolytic cleavage of pro-SigE pro-sequences (Dougan, 2013). The *Bacillus* SpoIIGA protein is involved in sporulation and septum formation (Fawcett et al., 2000). The reduced expression of SpoIIGA and other proteolysis genes such as sporulation protease and peptidase overtly hindered spore formation, since intermembrane proteolytic regulation is crucial during this stage. RNA polymerase factor sigma-70 (SigH), appeared to be the key regulatory switch, acting in the acidogenic phase. It may be a potential target gene for decreasing or preventing strain degeneration.

During acidogenesis, genes encoding acid producing enzymes showed higher expression levels in DG-8052. When acids accumulate to a certain level, CoA-transferase begins to re-assimilate them into cells and prepare solvent production. Indeed, decreased expression of CoA-transferase suppresses re-assimilation of acetic and butyric acids in *C. acetobutylicum* (Millat et al., 2014). The *sol* operon *ald*-*ctfA*-*ctfB, aadc* genes, were all down-regulated in DG-8052. The aldehyde dehydrogenase *aldA* gene is essential for the catabolism of alcohols, and regulated by sigma-54 in *Azotobacter vinelandii* (Gama-Castro et al., 2001). While, in *Clostridium kluyveri*, it was reported that acetoacetyl-CoA, 3-hydroxybutyryl-CoA and crotonyl-CoA are downregulated by Sigma-L, a member of the sigma54 family (Söhling and Gottschalk, 1996). In this study, a non-significant transcription level change of SigL may explain the similar transcription levels of acetoacetyl-CoA, 3-hydroxybutyryl-CoA and crotonyl-CoA in DG-8052.

Spore development (sporulation) is usually accompanied with solvent production in solventogenic *Clostridium* species (Lee et al., 2008). The sporulation associated sigma factors SigH, SigF, Sig K, SpoIIGA, SigE, and SigG were all down-regulated in DG-8052, leading to failed sporulation process. Since solventogenic genes are transcribed and expressed in the sporulation growth stage, uncompleted sporulation may result in reduced transcription of such genes. Therefore, the sigma-70 factor regulated spore formation may contribute to the lost capability of producing solvents in DG-8052 cells; CoA-transferase genes may be the main targets. Chemotaxis is a bacterial response to environmental stimuli. Motile bacteria migrate in smooth and straight lines toward attractants (positive chemotaxis) and tumble to avoid repellents (negative chemotaxis) (Gutierrez and Massox, 1987). The question raised by up-regulated chemotaxis genes in DG-8052 was how to identify potential attractants or repellents. In our previous report, fermentation of DG-8052 resulted in elevated concentrations of acetic and butyric acids as well as impaired production of acetone, butanol, and ethanol (Lv et al., 2016). Chemotaxis assays have shown that undissociated forms of acetic and butyric acids are attractants for *C. acetobutylicum*, with low pH alone not able to induce positive chemotaxis; meanwhile, acetone, butanol, ethanol, and dissociated acetate and butyrate were shown to be repellents (Gutierrez and Massox, 1987). The current findings are not only in line with previous reports, but also validate their conclusion at the mRNA level. In addition, the notion that undissociated acetic and butyric acids result in positive chemotaxis is also supported by another independent study demonstrating that *C. beijerinckii* challenged with furfural accumulates acetic and butyric acids and up-regulates chemotaxis genes (Zhang and Ezeji, 2013). The association of Spo0A and chemotaxis has been demonstrated by many studies. Overexpression of Spo0A in *C. acetobutylicum* results in decreased cell motility and chemotaxis (Alsaker et al., 2004); meanwhile, Spo0A inactivation in *C. acetobutylicum* SKO1 increases cell motility and chemotaxis genes (Tomas et al., 2003), corroborating this study that Spo0A down-regulation leads to increased transcription of chemotaxis genes.

DG-8052 cells could not complete the typical fermentation process, and died rapidly. It is possible that high concentrations of acetate and butyrate in the medium caused stress to DG-8052 cells. Such stress could induce weak metabolism and cause cell death in an early stage (Kohanski et al., 2008). ROS are formed when molecular oxygen diffuses into cells and is adventitiously reduced at the active sites of redox enzymes containing flavins or quinones. There is a pathway for H_2_O_2_ and O_2_ detoxification in *C. acetobutylicum* that includes NADH peroxidase and NADH oxidase, with detoxification activity depending on cell viability (Riebe et al., 2009). APF, a new ROS indicator, is non-fluorescent until it reacts with hydroxyl radicals, peroxynitrite anion or hypochlorite anion. Upon oxidation, APF exhibits bright green fluorescence, and was used to detect the hypochlorite anion generated by activated neutrophils (Setsukinai et al., 2003). Our findings on the decreased ability of ROS scavenging in DG-8052 could explain this phenomenon. DG-8052 cells had reduced viability compared with WT-8052 cells may also result from high oxidative stress. This suggests that ROS generation, bacterial cell morphology changes and cell degeneration are closely related. However, further study is needed to assess the effects of sigma factors on cell aging regulation.

During the growth of *C. beijerinckii* DG-8052, the accumulation of acetic and butyric acid led to the decreasing of pH (<5.5), and provided stress to the cell, which may cause the cell to die rapidly. The transcriptional analysis of non-solvent producing strain DG-8052 suggests morphological and physiological changes are related to altered expression of sigma factors. Dysregulation of the fermentation machinery which mainly including glycolysis to solvent/acid production, solventogenesis and sporulation, Chemotaxis, was accompanied by reduced transcription of rate-limiting enzymes *pfk* and *pyk* in glycolysis, and higher transcription of the cell chemotaxis genes *cheA, cheB, cheR, cheW*, and *cheY*, controlled mainly by σ^54^ in the acidogenesis phase. Meanwhile, abnormal spore formation was associated with repressed *spo0A, sigE, sigF, sigG*, and *sigK*, which were positively regulated by σ^70^ (*sigH*), with corresponding inhibited expression of CoA-transferase in the solventogenesis phase. And the specific gene targets need more experiment verification. These findings provide valuable targets for engineered strain development of *Clostridium* species.

## Author Contributions

BH, YZ, SJ, JL, and RD conducted the work presented here, performed data analysis and drafted the manuscript. All authors contributed to data interpretation, wrote and revised various parts of the paper. YZ, CW, RZ, and BH revised the overall paper; YZ and BH supervised the work. All authors read and approved the final manuscript.

## Conflict of Interest Statement

The authors declare that the research was conducted in the absence of any commercial or financial relationships that could be construed as a potential conflict of interest.

## Footnotes

1 https://earray.chem.agilent.com/earray/
