# Supplementary material for: Sigma Factor Regulated Cellular Response in a Non-solvent Producing Clostridium beijerinckii Degenerated Strain: A Comparative Transcriptome Analysis
Source: Front Microbiol. 2017 Jan 30;8:23. doi: 10.3389/fmicb.2017.00023 (PMC5276810; doi:10.3389/fmicb.2017.00023)
Supplement: Supplementary file 1 [file Table_1.PDF]

Table S1 List of 20 genes and sequences of primers used in Q-RT-PCR experiments for the validation of microarray analysis

Gene symbol	Gene product	Fold change		primer sequence	Length (bp)
		Microarray analysis	Q-RT-PCR		
Cbei_4123	heat shock protein Hsp20	+2.29*	+3.01*	F: 5′ AACCCAAACTCATTGGTGAA 3′<br>R: 5′ ATGTTTGGATTGATACCTTT 3′	115
Cbei_0311	electron transfer flavo protein subunit alpha-like protein	+3.64*	+5.21*	F: 5′ CGGTAGAACTGTAAGGCCAAG 3′<br>R: 5′ TGGTGCTTTATCATCAGCATT 3′	121
Cbei_3120	deoxyribose-phosphate aldolase	+19.41*	+24.98*	F: 5′ TGCTTGCTCCTGCATTTATC 3′<br>R: 5′ CTGGTGGAGCAACTAAGGAAG 3′	129
Cbei_0441	Permease (hypothetical protein)	+1.4*	+4.36*	F: 5′ TGGAGAGTCATTAGGAGGAACA 3′<br>R: 5′ TGAAGGTTCTTGAGTTTGCG 3′	124
Cbei_0331	inosine 5'-monophosphate dehydrogenase	+1.06*	+3.91*	F: 5′ GAAGGCCGTCTAAAGGGATT 3′<br>R: 5′ CACCTACTGCTGCTCCACAT 3′	112
Cbei_4824	response regulator receiver protein	+17.91*	+19.18*	F: 5′ AATGATCTTTATTGCTTCTATTCCC 3′<br>R: 5′ TGCGGATTTGAGATCATAGG 3′	135
Cbei_2740	phosphotransferase system,lactose/cellobiose-specific IIB subunit	+34.89*	+30.84*	F: 5′ TGCTGAGCAAGTTCCTTGAC 3′<br>R: 5′ TGAGGAAATATGCAGGGAAA3′	114
Cbei_4871	transketolase domain-containing protein	+13.09*	+13.21*	F: 5′ CAGACATTGATGCTTCCCAA 3′<br>R: 5′ AATGGCATTAGCAGGAAAGC 3′	104
Cbei_3356	methyl-accepting chemotaxis sensory transducer	+10.17*	+19.90*	F:5’ TGCTGAAGCTCTTGGTAGTGG 3’<br>R: 5’ TGCTGAAGCTCTTGGTAGTGG 3’	161
Cbei_3835	acetoacetate decarboxylase	-23.22*	-10.99*	F:5’ CAGCGTTTCCTAGAGGACCA 3’<br>R: 5’ CAGGCATAGCCATCATTTCA 3’	150
Cbei_1930	hypothetical protein	-6.61*	-6.07*	F: 5’ GGAAAAGCATGGTGCAAAA 3’<br>R: 5’ AGGATCTTCCTGTGCCATTG 3’	151

Cbei_0677	EmrB/QacA family drug resistance transporter	-18.02*	-23.23*	F: 5’ CGGAGTTTCCCATTCGTTTA 3’<br>R: 5’ CTGAGGCAATTATCCCTCCA 3’	203
Cbei_2826	carbohydrate-binding family V/XII protein	-9.81*	-14.12*	F: 5’ TCAATGGTGGAACAGTTGGA 3’<br>R: 5’ AGCGTCTTAGCAGGTTGTCC 3’	319
Cbei_2600	hypothetical protein	-4.46*	-19.35*	F: 5’ TGCGAATTAAGAAGCGATCC 3’<br>R: 5’ TGTTCACACCATTGCAGTCA 3’	181
Cbei_0411	acetyl-CoA acetyltransferase	+2.01**	+2.48**	F: 5′ TGTGGATCAGGTCTTAGAGCA 3′<br>R: 5′ CCCATCTTGCTTTGTCCAAT 3′	130
Cbei_1583	stage V sporulation protein E	-2.21**	-1.84**	F: 5’ GGAATTGGGGCTTATTGGAT 3’<br>R: 5’ TGGCATCGATCCTGTAACAA 3’	181
Cbei_0432	sigma 54 modulation protein	+1.93**	-1.03**	F: 5’ GAGGCGAAGAATCAACTTCC 3’<br>R: 5’ CCATCGTCCTCTTCTGATGG 3’	169
Cbei_0284	hypothetical protein	-18.66**	-24.18**	F: 5’ AGGTGAAAATCATTCCAAACG 3’<br>R: 5’ TCCGAGCTATCATTCTTTATAGGG 3’	192
Cbei_3832	aldehyde dehydrogenase	-26.35**	-30.55**	F: 5’ GTGGCGGTCCTGAAAATCTA 3’<br>R: 5’ TGATGCTCCTACCAGCCTTT 3’	225
Cbei_2261	lytic transglycosylase	-24.93**	-16.81**	F: 5’ CCAGCAAAACTTGCGAATCT 3’<br>R: 5’ CCATCACCAGATGAGGGACT 3’	197

*Fold change at 12h culture of *C. beijerinckii* DG8052; ** Fold change at 24h culture of *C. beijerinckii* DG8052

Table S2 Variants identified in *Clostridium beijerinckii* DG-8052[a] and their transcriptional change during fermentation

CDS region variants					
Gene ID	Product	Variant type	Coden	Fold change of genes by microarray analysis DG-8052 vs WT-8052	
				12h	24h
Cbei_0144	DNA-directed RNA polymerase subunit beta	A 182836 G	ACT→GCT	NC	NC
Cbei_0769	extracellular solute-binding protein	C 935449 T	CAA→TAA	+2.18	+3.28
Cbei_1046	hypothetical protein	T 1255087 C	GTA→GCA	NC	NC
Cbei_1069	2-nitropropane dioxygenase	T 1282245 C	GTT→GCT	NC	NC
Cbei_1685	hypothetical protein	G 1960764 T	AAG→AAT	ND	ND
Cbei_1854	peptidase S8/S53 subtilisin kexin sedolisin	T 2149286 C	GTT→GCT	NC	-4.35
Cbei_1933	glucose-inhibited division protein A	A 2232660 G	AAT→GAT	NC	NC
Cbei_1935	PucR family transcriptional regulator	C 2234607 A	CAT→AAT	NC	NC
Cbei_1975	adenine deaminase	G 2295776 T	GCT→TCT	NC	ND
Cbei_2569	DNA mismatch repair protein MutS	C 2979111 A	GCT→GAT	NC	-2.11
Cbei_2653	3-oxoacid CoA-transferase subunit B	C 3079373 T	GCT→GTT	NC	-36.19
Cbei_2769	extracellular ligand-binding receptor	G 3218907 A	GTG→ATG	NC	NC
Cbei_3078	PAS/PAC sensor hybrid histidine kinase	G 3591468 T	GAA→TAA	NC	NC
Cbei_3134	aldo/keto reductase	C 3665903 T	CCT→CTT	NC	NC
Cbei_4046	ethanolamine utilization protein EutJ family protein	A 4651590 G	ACT→GCT	ND	ND
Cbei_4400	Serine/threonine protein phosphatase-likeprotein	G 5075403 T	CGG→CTG	NC	NC
Cbei_4500	esterase	A 5205979 T	AAT→AAA	NC	NC
Cbei_4761	cell wall binding repeat-containing protein	T 5556196 C	GTT→GCT	NC	NC
Cbei_4905	glucose-1-phosphate adenylyltransferase	T 5755002 A	TTG→ATG	NC	NC
Cbei_1662	hypothetical protein	Ins ATCCGG after 1942328	frameshift	-5.28	-3.31
Cbei_4308	chemotaxis protein CheR	Del C after 4962014	frameshift	NC	NC
Cbei_2885	lactate permease	Ins T after 3369627	frameshift	+5.21	+3.71

Cbei_4998	ATPase	Ins A after 5883107	frameshift	NC	NC
Cbei_2830	glycoside hydrolase	Del CTACA after 3296490	frameshift	-9.94	-16.81
Cbei_0751	hypothetical protein	Ins T after 907959	frameshift	NC	NC
Cbei_2303	DNA-directed RNA polymerase sigma-70 factor	Del T after 2664570	frameshift	NC	NC
Cbei_0234	LacI family transcriptional regulator	Del AATAC after 266219	frameshift	NC	NC
Cbei_1790	single-stranded DNA-binding protein	Del A after 2074768	frameshift	-2.38	-2.30
Cbei_2386	fructose-6-phosphate aldolase	Del GAAGA after 2754381	frameshift	NC	NC
Intergenic region					

C 2611044 T	Intergenic of Cbei_2247 and Cbei_2248	No future found
Ins AG after 432413	Intergenic of Cbei_0356 and Cbei_0357	No future found
Del AA after 929926	Intergenic of Cbei_R0070 and Cbei_0765	No future found
Del TT after 1702123	Intergenic of Cbei_1445 and Cbei_1446	No future found
Ins TG after 2399098	Intergenic of Cbei_2060 and Cbei_2061	No future found
Del TTT after 3043073	Intergenic of Cbei_2618 and Cbei_2619	No future found
Del TATCTC after 5382086	Intergenic of Cbei_4644 and Cbei_4645	No future found

[a] single nucleotide variations (SNVs) and InDel were identified by aligning the sequenced data with the reference genome of *C. beijerinckii* NCIMB8052.

* ND, not detected, NC, no changed.

Table S3a Differentially expressed (upregulation) genes in DG-8052 vs WT-8052 during acidogenic phase

GOTERM_ Category	GO number	Term	Count	%	P-value
Biological Process	0007165	signal transduction	45	8.46	5.92E-07
	0005975	carbohydrate metabolic process	60	11.28	2.06E-06
	0000160	two-component signal transduction system (phosphorelay)	42	7.90	3.46E-06
	0007626	locomotory behavior	24	4.51	6.31E-05
	0042330	taxis	24	4.51	6.31E-05
	0006935	chemotaxis	24	4.51	6.31E-05
	0007610	behavior	24	4.51	6.31E-05
	0008643	carbohydrate transport	34	6.39	7.05E-05
	0009401	phosphoenolpyruvate-dependent sugar phosphotransferase system	32	6.02	9.26E-05
	0065007	biological regulation	106	19.92	1.84E-04
	0050794	regulation of cellular process	104	19.55	1.96E-04
	0044262	cellular carbohydrate metabolic process	27	5.075	2.45E-04
	0050789	regulation of biological process	104	19.55	2.92E-04
	0040011	locomotion	24	4.51	4.19E-04
	0009605	response to external stimulus	24	4.51	6.87E-04
	0006066	alcohol metabolic process	23	4.32	7.81E-04
	0018202	peptidyl-histidine modification	21	3.95	0.001
	0018106	peptidyl-histidine phosphorylation	21	3.95	0.001
	0018193	peptidyl-amino acid modification	21	3.95	0.002
	0042221	response to chemical stimulus	28	5.26	0.002
	0006468	protein amino acid phosphorylation	21	3.95	0.002
	0009056	catabolic process	24	4.51	0.003
	0006464	protein modification process	23	4.32	0.003
	0016310	phosphorylation	25	4.70	0.004
	0005996	monosaccharide metabolic process	19	3.57	0.005
	0006796	phosphate metabolic process	26	4.89	0.005
	0006793	phosphorus metabolic process	26	4.89	0.006
	0043687	post-translational protein modification	21	3.95	0.008
	0016052	carbohydrate catabolic process	16	3.01	0.014
	0019318	hexose metabolic process	14	2.63	0.019

	0044248	cellular catabolic process	10	1.88	0.021
	0044275	cellular carbohydrate catabolic process	11	2.07	0.022
	0009166	nucleotide catabolic process	4	0.75	0.027
	0043412	biopolymer modification	24	4.51	0.027
	0006020	inositol metabolic process	3	0.56	0.041
	0019310	inositol catabolic process	3	0.56	0.041
Cellular Component	0016021	integral to membrane	63	11.84	0.014
	0031224	intrinsic to membrane	63	11.84	0.016
	0044425	membrane part	64	12.03	0.025
Molecular Function	0060089	molecular transducer activity	67	12.59	1.74E-09
	0004871	signal transducer activity	67	12.59	1.74E-09
	0016773	phosphotransferase activity, alcohol group as acceptor	50	9.40	8.20E-07
	0016772	transferase activity, transferring phosphorus-containing groups	63	11.84	1.62E-05
	0015144	carbohydrate transmembrane transporter activity	27	5.08	2.55E-04
	0008982	protein-N(PI)-phosphohistidine-sugar phosphotransferase activity	18	3.38	2.97E-04
	0000155	two-component sensor activity	25	4.70	9.83E-04
	0004673	protein histidine kinase activity	25	4.70	9.83E-04
	0000156	two-component response regulator activity	24	4.51	0.001
	0016775	phosphotransferase activity, nitrogenous group as acceptor	25	4.70	0.001
	0051119	sugar transmembrane transporter activity	23	4.32	0.001
	0016301	kinase activity	40	7.52	0.002
	0004672	protein kinase activity	25	4.70	0.003
	0005402	cation:sugar symporter activity	20	3.76	0.004
	0050660	FAD binding	13	2.44	0.005
	0015295	solute:hydrogen symporter activity	19	3.57	0.007
	0005351	sugar:hydrogen symporter activity	19	3.57	0.007
	0016758	transferase activity, transferring hexosyl groups	8	1.50	0.009
	0015294	solute:cation symporter activity	20	3.76	0.009
	0015293	symporter activity	20	3.76	0.009
	0016861	intramolecular oxidoreductase activity, interconverting aldoses and ketoses	8	1.50	0.012

	0016860	intramolecular oxidoreductase activity	8	1.50	0.024
	0005529	sugar binding	8	1.50	0.036
	0030246	carbohydrate binding	10	1.88	0.038
	0016853	isomerase activity	20	3.76	0.043

Table S3b Differentially expressed (downregulation) genes in DG-8052 vs WT-8052 during acidogenic phase

GOTERM _Category	GO number	Term	Count	%	P-value
Biological Process	0009081	branched chain family amino acid metabolic process	7	1.08	0.001
	0009082	branched chain family amino acid biosynthetic process	7	1.08	0.001
	0006551	leucine metabolic process	4	0.62	0.011
	0009098	leucine biosynthetic process	4	0.62	0.011
	0009309	amine biosynthetic process	18	2.77	0.043
	0006810	transport	81	12.48	2.04E-05
	0051234	establishment of localization	81	12.48	2.04E-05
	0051179	localization	81	12.48	7.63E-05
	0008272	sulfate transport	5	0.77	0.002
	0015850	organic alcohol transport	6	0.92	0.013
	0015904	tetracycline transport	6	0.92	0.013
	0042891	antibiotic transport	6	0.92	0.013
	0032324	molybdopterin cofactor biosynthetic process	5	0.77	0.011
	0051189	prosthetic group metabolic process	5	0.77	0.011
	0043545	molybdopterin cofactor metabolic process	5	0.77	0.011
	0006777	Mo-molybdopterin cofactor biosynthetic process	5	0.77	0.011
	0019720	Mo-molybdopterin cofactor metabolic process	5	0.77	0.011
	0019438	aromatic compound biosynthetic process	12	1.85	0.018
	0006725	cellular aromatic compound metabolic process	16	2.47	0.024
	0042558	pteridine and derivative metabolic process	5	0.77	0.043
	0042559	pteridine and derivative biosynthetic process	5	0.77	0.043
Cellular Component	0016020	membrane	104	16.02	0.011
	0016021	integral to membrane	68	10.48	0.046
	0030313	cell envelope	13	2.00	0.003

	0031975	envelope	13	2.00	0.004
	0030312	external encapsulating structure	13	2.00	0.006
	0044462	external encapsulating structure part	7	1.08	0.043
	0005576	extracellular region	15	2.31	0.047
Molecular Function	0005215	transporter activity	66	10.17	0.001
	0022892	substrate-specific transporter activity	41	6.32	0.016
	0008509	anion transmembrane transporter activity	8	1.23	0.010
	0015116	sulfate transmembrane transporter activity	4	0.62	0.012
	0015520	tetracycline:hydrogen antiporter activity	6	0.93	0.014
	0008493	tetracycline transporter activity	6	0.93	0.014
	0042895	antibiotic transporter activity	6	0.93	0.014
	0015307	drug: hydrogen antiporter activity	6	0.93	0.014
	0015300	solute:solute antiporter activity	6	0.93	0.026
	0015298	solute:cation antiporter activity	6	0.93	0.026
	0015299	solute:hydrogen antiporter activity	6	0.93	0.026
	0016462	pyrophosphatase activity	40	6.16	0.044

Table S3c Differentially expressed (upregulation) genes in DG-8052 vs WT-8052 during solventogenic phase

GOTERM_ Category	GO number	Term	Count	%	P-value
Biological Process	0005975	carbohydrate metabolic process	66	9.66	1.28E-04
	0044262	cellular carbohydrate metabolic process	30	4.39	0.001
	0006066	alcohol metabolic process	26	3.81	0.002
	0005996	monosaccharide metabolic process	22	3.22	0.007
	0016052	carbohydrate catabolic process	19	2.78	0.014
	0009056	catabolic process	24	3.51	0.048
	0065007	biological regulation	142	20.79	6.28E-07
	0050794	regulation of cellular process	139	20.35	8.71E-07
	0050789	regulation of biological process	139	20.35	1.61E-06
	0006355	regulation of transcription, DNA-dependent	90	13.18	0.005
	0051252	regulation of RNA metabolic process	90	13.18	0.005
	0045449	regulation of transcription	92	13.47	0.006
	0051171	regulation of nitrogen compound metabolic process	92	13.47	0.006
	0019219	regulation of nucleobase, nucleoside, nucleotide and nucleic acid metabolic process	92	13.47	0.006
	0009889	regulation of biosynthetic process	92	13.47	0.007
	0010556	regulation of macromolecule biosynthetic process	92	13.47	0.007
	0031326	regulation of cellular biosynthetic process	92	13.47	0.007
	0010468	regulation of gene expression	92	13.47	0.008
	0031323	regulation of cellular metabolic process	92	13.47	0.008
	0080090	regulation of primary metabolic process	92	13.47	0.008
	0060255	regulation of macromolecule metabolic process	92	13.47	0.009
	0019222	regulation of metabolic process	92	13.47	0.010
	0016310	phosphorylation	28	4.10	0.014
	0043412	biopolymer modification	30	4.39	0.017

	0006796	phosphate metabolic process	29	4.25	0.019
	0006793	phosphorus metabolic process	29	4.25	0.022
	0018202	peptidyl-histidine modification	23	3.37	0.005
	0018106	peptidyl-histidine phosphorylation	23	3.37	0.005
	0018193	peptidyl-amino acid modification	23	3.37	0.007
	0006468	protein amino acid phosphorylation	23	3.37	0.008
	0006464	protein modification process	29	4.25	8.07E-04
	0043687	post-translational protein modification	27	3.95	0.001766
	0007165	signal transduction	55	8.05	9.72E-08
	0009401	phosphoenolpyruvate-dependent sugar phosphotransferase system	43	6.29	7.10E-07
	0008643	carbohydrate transport	44	6.44	2.48E-06
	0000160	two-component signal transduction system (phosphorelay)	51	7.47	1.06E-06
	0007165	signal transduction	55	8.05	9.72E-08
	0007610	behavior	31	4.54	2.04E-06
	0042330	taxis	31	4.54	2.04E-06
	0007626	locomotory behavior	31	4.54	2.04E-06
	0006935	chemotaxis	31	4.54	2.04E-06
	0009605	response to external stimulus	32	4.69	2.11E-05
	0040011	locomotion	31	4.54	2.93E-05
	0042221	response to chemical stimulus	35	5.12	5.87E-04
	0050896	response to stimulus	41	6.00	0.067465
Molecular Function	0016758	transferase activity, transferring hexosyl groups	8	1.17	0.034
	0060089	molecular transducer activity	83	12.15	6.62E-11
	0004871	signal transducer activity	83	12.15	6.62E-11
	0015144	carbohydrate transmembrane transporter activity	31	4.54	5.76E-04
	0015295	solute:hydrogen symporter activity	24	3.51	0.002
	0005351	sugar:hydrogen symporter activity	24	3.51	0.002
	0005402	cation:sugar symporter activity	24	3.51	0.003
	0015293	symporter activity	25	3.66	0.004
	0015294	solute:cation symporter activity	25	3.66	0.004
	0051119	sugar transmembrane transporter activity	26	3.81	0.004

	0016798	hydrolase activity, acting on glycosyl bonds	23	3.37	0.002
	0004553	hydrolase activity, hydrolyzing O-glycosyl compounds	20	2.93	0.007
	0000156	two-component response regulator activity	30	4.40	3.17E-04
	0004871	signal transducer activity	83	12.15	6.62E-11
	0060089	molecular transducer activity	83	12.15	6.62E-11
	0008982	protein-N(PI)-phosphohistidine-sugar phosphotransferase activity	25	3.66	1.66E-06
	0008324	cation transmembrane transporter activity	26	3.81	0.385
	0022892	substrate-specific transporter activity	43	6.30	0.397
	0005215	transporter activity	67	9.81	0.427
	0022857	transmembrane transporter activity	43	6.30	0.452
	0016773	phosphotransferase activity, alcohol group as acceptor	60	8.78	5.32E-07
	0015291	secondary active transmembrane transporter activity	29	4.25	0.052
	0022891	substrate-specific transmembrane transporter activity	42	6.15	0.147
	0022804	active transmembrane transporter activity	40	5.86	0.203
	0015075	ion transmembrane transporter activity	32	4.69	0.259
	0016772	transferase activity, transferring phosphorus-containing groups	73	10.69	1.32E-04
	0016301	kinase activity	48	7.03	0.003
	0004672	protein kinase activity	27	3.95	0.020
	0030528	transcription regulator activity	79	11.57	0.013
	0005215	transporter activity	67	9.81	0.427
	0000155	two-component sensor activity	26	3.81	0.014
	0004673	protein histidine kinase activity	26	3.81	0.014
	0016775	phosphotransferase activity, nitrogenous group as acceptor	26	3.81	0.016
Cellular Component	0044464	cell part	197	28.84	0.502
	0005623	cell	197	28.84	0.502

	0016021	integral to membrane	87	12.74	4.60E-04
	0031224	intrinsic to membrane	87	12.74	5.50E-04
	0044425	membrane part	88	12.88	0.001
	0016020	membrane	116	16.98	0.021

Table S3d Differentially expressed (down-regulation) genes in DG8052 vs WT-8052 during solventogenic phase

GOTERM_ Category	GO number	Term	Count	%	P-value
Biological Process	0032502	developmental process	9	1.22	1.91E-04
	0043934	sporulation	6	0.81	0.003
	0009653	anatomical structure morphogenesis	6	0.81	0.009
	0048856	anatomical structure development	6	0.81	0.009
	0048869	cellular developmental process	6	0.81	0.009
	0030154	cell differentiation	5	0.68	0.015
	0030435	sporulation resulting in formation of a cellular spore	5	0.68	0.015
	0048646	anatomical structure formation involved in morphogenesis	5	0.68	0.015
	0006508	proteolysis	19	2.58	0.004
	0019538	protein metabolic process	44	5.97	0.177
	0006022	aminoglycan metabolic process	10	1.36	0.029
	0006351	transcription, DNA-dependent	10	1.36	0.04
	0032774	RNA biosynthetic process	10	1.36	0.046
	0006265	DNA topological change	7	0.95	0.018
	0015893	drug transport	9	1.22	0.016
	0042493	response to drug	9	1.22	0.016
Cellular Component	0005576	extracellular region	4	0.54	0.04
Molecular Function	0070011	peptidase activity, acting on L-amino acid peptides	20	2.71	0.004
	0004175	endopeptidase activity	13	1.76	0.006
	0008233	peptidase activity	21	2.85	0.007
	0008237	metallopeptidase activity	8	1.09	0.166
	0046914	transition metal ion binding	44	5.97	0.003
	0046872	metal ion binding	58	7.87	0.01
	0051539	4 iron, 4 sulfur cluster binding	18	2.44	0.013
	0043169	cation binding	58	7.87	0.017
	0043167	ion binding	58	7.87	0.017
	0009055	electron carrier activity	29	3.93	0.018
	0051536	iron-sulfur cluster binding	26	3.53	0.032
	0051540	metal cluster binding	26	3.53	0.032
	0005506	iron ion binding	21	2.85	0.037
	0043566	structure-specific DNA binding	9	1.22	0.006
	0015297	antiporter activity	9	1.22	0.044

	0003690	double-stranded DNA binding	7	0.95	0.023
	0030246	carbohydrate binding	8	1.09	0.202
	0016798	hydrolase activity, acting on glycosyl bonds	11	1.49	0.472
	0004553	hydrolase activity, hydrolyzing O-glycosyl compounds	10	1.36	0.49
	0030976	thiamin pyrophosphate binding	5	0.68	0.038
	0015238	drug transporter activity	9	1.22	0.021

Table S4a Genes differentially expressed by more than 5 folds in DG-8052 vs WT-8052 during acidogenic phase

Fold Change	up/ down	Gene Symbol	Gene Product
5.03	up	Cbei_4509	glycerol kinase
5.05	up	Cbei_2967	hypothetical protein
5.09	up	Cbei_2952	hypothetical protein
5.09	up	Cbei_2212	integral membrane protein TerC
5.12	up	Cbei_2971	NLP/P60 protein
5.13	up	Cbei_0346	nitroreductase
5.16	up	Cbei_0804	methyl-accepting chemotaxis sensory transducer
5.17	up	Cbei_4832	methyl-accepting chemotaxis sensory transducer
5.17	up	Cbei_4554	HAD family hydrolase
5.18	up	Cbei_0583	membrane protein
5.21	up	Cbei_2885	L-lactate transport
5.29	up	Cbei_2495	hypothetical protein
5.37	up	Cbei_0245	mannitol dehydrogenase domain-containingprotein
5.41	up	Cbei_2968	DoxX family protein
5.58	up	Cbei_4465	aldose 1-epimerase
5.6	up	Cbei_2248	ribosomal RNA adenine dimethylase
5.61	up	Cbei_0965	PTS system sorbose-specific transporter subunitIIC
5.63	up	Cbei_0862	PucR family transcriptional regulator
5.66	up	Cbei_2753	glycerol dehydrogenase
5.69	up	Cbei_2290	hypothetical protein
5.69	up	Cbei_2983	---
5.75	up	Cbei_0595	RNA polymerase factor sigma-54
5.82	up	Cbei_2458	5-keto-4-deoxyuronate isomerase
5.87	up	Cbei_0244	"phosphoenolpyruvate-dependent sugarphosphotransferase system, EIIA 2"
5.96	up	Cbei_3470	hypothetical protein
6	up	Cbei_2982	hypothetical protein
6.05	up	Cbei_0664	alpha amylase
6.06	up	Cbei_4095	dihydroxy-acid dehydratase
6.13	up	Cbei_2384	xylulokinase
6.15	up	Cbei_2841	SCP-like extracellular protein
6.16	up	Cbei_1236	glycoside hydrolase
6.21	up	Cbei_1510	dihydroxy-acid dehydratase
6.3	up	Cbei_3325	ABC transporter
6.31	up	Cbei_0861	ABC transporter
6.34	up	Cbei_0243	transcriptional antiterminator BglG
6.36	up	Cbei_4555	sigma-54 factor interaction domain-containingprotein
6.38	up	Cbei_1432	hypothetical protein
6.39	up	Cbei_2160	multi-sensor hybrid histidine kinase
6.54	up	Cbei_3323	antibiotic transport-associated permeaseSpaG/MutG
6.72	up	Cbei_0735	alpha amylase
6.78	up	Cbei_2284	hydroxylamine reductase

6.8	up	Cbei_3778	glycosyl transferase family protein
6.98	up	Cbei_3968	hypothetical protein
7.11	up	Cbei_0285	glycogen/starch/alpha-glucan phosphorylase
7.21	up	Cbei_0312	FAD linked oxidase domain-containing protein
7.23	up	Cbei_3472	oxidoreductase FAD/NAD(P)-binding subunit
7.38	up	Cbei_3471	signal transduction protein
7.4	up	Cbei_2843	hypothetical protein
7.51	up	Cbei_4450	monosaccharide-transporting ATPase
7.54	up	Cbei_4511	MIP family channel protein
7.55	up	Cbei_2997	hypothetical protein
7.84	up	Cbei_3324	lantibiotic permease spaE/mutE
7.88	up	Cbei_3469	histidine kinase
7.9	up	Cbei_4546	xylose isomerase domain-containing protein
8.11	up	Cbei_0473	hypothetical protein
8.22	up	Cbei_0966	PTS system mannose/fructose/sorbose familytransporter subunit IID
8.25	up	Cbei_0734	sugar ABC transporter permease
8.29	up	Cbei_2998	hypothetical protein
8.39	up	Cbei_3986	SCP-like extracellular protein
8.53	up	Cbei_3023	hypothetical protein
8.56	up	Cbei_2849	methyl-accepting chemotaxis sensory transducer
8.6	up	Cbei_0732	extracellular solute-binding protein
8.87	up	Cbei_1855	hypothetical protein
8.93	up	Cbei_3287	---
9.26	up	Cbei_2488	hypothetical protein
9.27	up	Cbei_4469	SCP-like extracellular protein
9.29	up	Cbei_0963	PTS system fructose subfamily transporter subunit IIA
9.34	up	Cbei_4547	oxidoreductase domain-containing protein
9.34	up	Cbei_3122	inner-membrane translocator
9.45	up	Cbei_0233	4-alpha-glucanotransferase
9.58	up	Cbei_3014	hydroxylamine reductase
9.69	up	Cbei_0964	PTS system sorbose subfamily transporter subunit IIB
10	up	Cbei_0242	PTS system mannitol-specific transporter subunit IIC
10	up	Cbei_2842	hypothetical protein
10.1	up	Cbei_4718	cell wall binding repeat-containing protein
10.1	up	Cbei_0733	sugar ABC transporter permease
10.1	up	Cbei_2844	ABC transporter
10.1	up	Cbei_2039	sigma-54 dependent transcriptional regulator
10.2	up	Cbei_3356	methyl-accepting chemotaxis sensory transducer
10.7	up	Cbei_1723	methyl-accepting chemotaxis sensory transducer
10.9	up	Cbei_4828	methyl-accepting chemotaxis sensory transducer
11.1	up	Cbei_4829	chemotaxis protein CheA
11.1	up	Cbei_4819	response regulator receiver protein
11.4	up	Cbei_1713	hemerythrin-like metal-binding protein
11.5	up	Cbei_4670	6-phospho-beta-glucosidase
11.9	up	Cbei_2489	hypothetical protein

12.1	up	Cbei_4549	myo-inositol catabolism IolB domain-containing protein
12.4	up	Cbei_4688	PAS/PAC sensor-containing diguanylate cyclase
12.5	up	Cbei_4827	protein-glutamate O-methyltransferase
13.1	up	Cbei_4871	transketolase domain-containing protein
13.4	up	Cbei_4550	ribokinase-like domain-containing protein
13.6	up	Cbei_3625	methyl-accepting chemotaxis sensory transducer
13.6	up	Cbei_3130	GntR family transcriptional regulator
14.3	up	Cbei_0967	sugar isomerase (SIS)
14.4	up	Cbei_4816	hemerythrin-like metal-binding protein
14.5	up	Cbei_2348	hypothetical protein
15	up	Cbei_2169	hypothetical protein
15.1	up	Cbei_4548	"thiamine pyrophosphate protein, central region"
15.2	up	Cbei_4551	fructose-bisphosphate aldolase
15.3	up	Cbei_4820	response regulator receiver sensor signal transduction histidine kinase
15.5	up	Cbei_3288	metal dependent phosphohydrolase
16.4	up	Cbei_4180	response regulator receiver modulated CheB methylesterase
16.6	up	Cbei_2787	methyl-accepting chemotaxis sensory transducer
16.9	up	Cbei_4181	protein-glutamate O-methyltransferase
17.6	up	Cbei_4818	hemerythrin-like metal-binding protein
17.7	up	Cbei_2725	response regulator receiver sensor signal transduction histidine kinase
17.9	up	Cbei_4824	response regulator receiver protein
18.8	up	Cbei_0232	binding-protein-dependent transport system inner membrane protein
19.4	up	Cbei_3120	deoxyribose-phosphate aldolase
19.9	up	Cbei_2738	transcriptional anti-terminator BglG
20	up	Cbei_4552	iron-containing alcohol dehydrogenase
20.3	up	Cbei_3129	uridine phosphorylase
20.4	up	Cbei_4822	CheW protein
20.8	up	Cbei_4817	hypothetical protein
20.9	up	Cbei_4183	chemotaxis protein CheA
21.2	up	Cbei_2728	PAS/PAC sensor signal transduction histidine kinase
21.4	up	Cbei_4821	methyl-accepting chemotaxis sensory transducer
22.1	up	Cbei_4558	PTS system sorbose-specific transporter subunit IIC
22.8	up	Cbei_4823	methyl-accepting chemotaxis sensory transducer
23.6	up	Cbei_3126	hypothetical protein
24	up	Cbei_0782	formate/nitrite transporter
24	up	Cbei_0231	binding-protein-dependent transport system inner membrane protein
24.3	up	Cbei_3407	cell wall binding repeat-containing protein
24.6	up	Cbei_4560	PTS system fructose subfamily transporter subunit IIA
25.7	up	Cbei_2727	signal transduction protein
25.9	up	Cbei_3128	cytidine deaminase
26.6	up	Cbei_3127	phosphopentomutase

27.6	up	Cbei_2850	methyl-accepting chemotaxis protein
28.3	up	Cbei_4556	sugar isomerase (SIS)
28.7	up	Cbei_2726	response regulator receiver protein
28.9	up	Cbei_2167	hypothetical protein
30	up	Cbei_2166	hypothetical protein
30.4	up	Cbei_3124	basic membrane lipoprotein
33.8	up	Cbei_2165	hemerythrin-like metal-binding protein
34.9	up	Cbei_2740	"phosphotransferase system, lactose / cellobiose-specific IIB subunit"
36.2	up	Cbei_2170	hypothetical protein
36.9	up	Cbei_4182	methyl-accepting chemotaxis sensory transducer
38.9	up	Cbei_4559	PTS system sorbose subfamily transporter subunit IIB
39.2	up	Cbei_2742	translaldolase
39.6	up	Cbei_2168	hypothetical protein
41.6	up	Cbei_3755	hypothetical protein
41.9	up	Cbei_0784	anaerobic sulfite reductase subunit B
42.3	up	Cbei_4557	PTS system mannose/fructose/sorbose family transporter subunit IID
45.6	up	Cbei_0230	extracellular solute-binding protein
46.4	up	Cbei_0783	sulfite reductase subunit A
47.2	up	Cbei_0785	sulfite reductase subunit C
50.1	up	Cbei_2739	PTS system ascorbate-specific transporter subunit IIC
59.4	up	Cbei_4184	CheW protein
62.4	up	Cbei_2741	PTS transporter subunit IIA-like nitrogen-regulatory protein PtsN
63.8	up	Cbei_3125	purine nucleoside phosphorylase
5.03	down	Cbei_3916	major facilitator superfamily transporter
5.1	down	Cbei_0519	serine-type D-Ala-D-Ala carboxy peptidase
5.14	down	Cbei_2774	cell wall binding repeat-containing protein
5.14	down	Cbei_3794	formate dehydrogenase accessory protein FdhD
5.17	down	Cbei_1001	aspartate carbamoyl transferase regulatory subunit
5.26	down	Cbei_3796	"hydrogenase, Fe-only"
5.27	down	Cbei_2731	NADH: flavin oxidoreductase
5.28	down	Cbei_1662	hypothetical protein
5.29	down	Cbei_3723	hypothetical protein
5.31	down	Cbei_4363	hypothetical protein
5.33	down	Cbei_0676	transposase
5.33	down	Cbei_1561	hypothetical protein
5.35	down	Cbei_1558	hypothetical protein
5.35	down	Cbei_1657	hypothetical protein
5.39	down	Cbei_2321	"alpha, alpha-phosphotrehalase"
5.43	down	Cbei_3207	cell wall binding repeat-containing protein
5.44	down	Cbei_0592	VanW family protein
5.46	down	Cbei_3113	hypothetical protein
5.46	down	Cbei_0150	30S ribosomal protein S10
5.47	down	Cbei_4159	LuxR family transcriptional regulator
5.49	down	Cbei_1988	molybdopterin binding domain-containing protein

5.5	down	Cbei_0212	acetolactate synthase small subunit
5.5	down	Cbei_3034	response regulator receiver protein
5.57	down	Cbei_0508	50S ribosomal protein L21
5.58	down	Cbei_3759	FAD linked oxidase domain-containing protein
5.65	down	Cbei_3685	hypothetical protein
5.69	down	Cbei_4364	hypothetical protein
5.73	down	Cbei_4193	sulfate ABC transporter substrate-binding protein
5.74	down	Cbei_3684	ATPase AAA
5.79	down	Cbei_2829	RNA polymerase sigma factor SigI
5.8	down	Cbei_4157	radical SAM domain-containing protein
5.87	down	Cbei_3021	hypothetical protein
5.89	down	Cbei_3800	molybdenum cofactor synthesis domain-containing protein
6.01	down	Cbei_0247	serine O-acetyltransferase
6.07	down	Cbei_0248	oleoyl-(acyl-carrier-protein) hydrolase
6.15	down	Cbei_2126	aspartyl-tRNA synthetase
6.21	down	Cbei_3686	hypothetical protein
6.22	down	Cbei_4583	hypothetical protein
6.24	down	Cbei_1559	uridine kinase
6.24	down	Cbei_1000	aspartate carbamoyltransferase catalytic subunit
6.33	down	Cbei_4581	---
6.54	down	Cbei_4700	hypothetical protein
6.57	down	Cbei_5042	extracellular ligand-binding receptor
6.6	down	Cbei_4221	cell wall binding repeat-containing protein
6.61	down	Cbei_1930	hypothetical protein
6.62	down	Cbei_1606	fibronectin type III domain-containing protein
6.66	down	Cbei_4079	hypothetical protein
6.77	down	Cbei_0358	hypothetical protein
6.86	down	Cbei_1008	hypothetical protein
6.86	down	Cbei_3798	formate dehydrogenase family accessory protein FdhD
6.89	down	Cbei_3964	CHAP domain-containing protein
6.97	down	Cbei_3801	formate dehydrogenase subunit alpha
7.09	down	Cbei_3762	transport system permease
7.18	down	Cbei_4365	hypothetical protein
7.4	down	Cbei_0250	amino acid adenylation domain-containing protein
7.46	down	Cbei_3256	hypothetical protein
7.47	down	Cbei_3799	molybdopterin-guanine dinucleotide biosynthesis protein MobB
7.48	down	Cbei_2828	glycoside hydrolase
7.52	down	Cbei_1882	hypothetical protein
7.67	down	Cbei_2583	hypothetical protein
7.7	down	Cbei_3797	4Fe-4S ferredoxin
7.98	down	Cbei_1826	RNA polymerase sigma factor SigI
8.02	down	Cbei_3168	phosphoglycerate mutase
8.03	down	Cbei_4023	cell wall hydrolase/autolysin
8.05	down	Cbei_2827	---
8.44	down	Cbei_3167	hypothetical protein
8.64	down	Cbei_0948	hypothetical protein

9.03	down	Cbei_2127	glutamyl-tRNA(Gln) amidotransferase subunit C
9.06	down	Cbei_1061	hypothetical protein
9.37	down	Cbei_3283	hypothetical protein
9.49	down	Cbei_3170	hypothetical protein
9.5	down	Cbei_3043	hypothetical protein
9.68	down	Cbei_3834	3-oxoacid CoA-transferase subunit B
9.81	down	Cbei_2826	carbohydrate-binding family V/XII protein
9.94	down	Cbei_2830	glycoside hydrolase
10.1	down	Cbei_0691	acyl-ACP thioesterase
10.3	down	Cbei_3923	integral membrane sensor signal transduction histidine kinase
10.4	down	Cbei_2587	triple helix repeat-containing collagen
10.6	down	Cbei_4584	ABC transporter
10.8	down	Cbei_0688	beta-lactamase domain-containing protein
11	down	Cbei_3171	accessory gene regulator B
11	down	Cbei_3688	hypothetical protein
11.1	down	Cbei_2831	carbohydrate-binding family V/XII protein
11.5	down	Cbei_0689	hypothetical protein
11.8	down	Cbei_0282	hypothetical protein
11.9	down	Cbei_3836	"peptidase C1A, papain"
12.1	down	Cbei_1863	hypothetical protein
12.1	down	Cbei_0283	ABC transporter
12.1	down	Cbei_3169	multi-sensor signal transduction histidine kinase
12.2	down	Cbei_3197	glycosyl transferase family protein
13	down	Cbei_0284	hypothetical protein
13.4	down	Cbei_0281	hypothetical protein
13.9	down	Cbei_3268	"holin, phage phi LC3"
13.9	down	Cbei_0682	hypothetical protein
14.1	down	Cbei_0690	radical SAM domain-containing protein
14.1	down	Cbei_4585	NADPH-dependent FMN reductase
14.3	down	Cbei_3833	3-oxoacid CoA-transferase subunit A
14.5	down	Cbei_2071	hypothetical protein
16.4	down	Cbei_2584	hypothetical protein
16.6	down	Cbei_3687	hypothetical protein
17.2	down	Cbei_3832	aldehyde dehydrogenase
17.7	down	Cbei_2261	lytic transglycosylase
17.8	down	Cbei_0280	hypothetical protein
18	down	Cbei_0677	EmrB/QacA family drug resistance transporter
18.3	down	Cbei_5022	hypothetical protein
18.9	down	Cbei_0684	AMP-dependent synthetase and ligase
19.4	down	Cbei_1888	hypothetical protein
20.6	down	Cbei_3495	beta-lactamase domain-containing protein
22	down	Cbei_0687	4'-phosphopantetheinyl transferase
22.1	down	Cbei_0686	AMP-dependent synthetase and ligase
22.3	down	Cbei_4586	lantibiotic modifying -like protein
22.7	down	Cbei_3049	ABC transporter
23.2	down	Cbei_3835	acetoacetate decarboxylase
24.9	down	Cbei_0685	alcohol dehydrogenase
26.2	down	Cbei_3048	---

29.5	down	Cbei_0681	thioesterase
31.6	down	Cbei_0683	radical SAM domain-containing protein
32.2	down	Cbei_0679	ABC transporter
33.4	down	Cbei_4587	hypothetical protein
35	down	Cbei_0673	---
41.8	down	Cbei_0680	O-methyltransferase family protein
50.6	down	Cbei_0674	NAD-dependent aldehyde dehydrogenase-like protein
59.4	down	Cbei_0675	coenzyme F390 synthetase-like protein

Table S4b Genes differentially expressed by more than 5 folds in DG-8052 vs WT-8052 during solventogenic phase

Fold Change	up/ down	Gene Symbol	Gene Product
5.03	up	Cbei_1932	iron-containing alcohol dehydrogenase
5.13	up	Cbei_4008	malate dehydrogenase
5.14	up	Cbei_0861	ABC transporter
5.14	up	Cbei_4479	glycerate kinase
5.16	up	Cbei_3671	methyl-accepting chemotaxis sensory transducer
5.2	up	Cbei_4815	hypothetical protein
5.22	up	Cbei_2981	hypothetical protein
5.23	up	Cbei_3320	methyl-accepting chemotaxis sensory transducer
5.24	up	Cbei_2849	methyl-accepting chemotaxis sensory transducer
5.38	up	Cbei_2952	hypothetical protein
5.48	up	Cbei_2495	hypothetical protein
5.48	up	Cbei_1727	UTP-glucose-1-phosphate uridylyltransferase
5.5	up	Cbei_4002	endopeptidase Clp
5.52	up	Cbei_3777	small multidrug resistance transmembrane protein
5.57	up	Cbei_0346	nitroreductase
5.6	up	Cbei_3819	coenzyme A transferase
5.6	up	Cbei_3322	two component transcriptional regulator
5.61	up	Cbei_4719	cell wall binding repeat-containing protein
5.66	up	Cbei_1726	hypothetical protein
5.69	up	Cbei_1433	hypothetical protein
5.7	up	Cbei_2290	hypothetical protein
5.71	up	Cbei_1510	dihydroxy-acid dehydratase
5.76	up	Cbei_2871	glycine/betaine ABC transporter ATPase
5.78	up	Cbei_2983	---
5.88	up	Cbei_2738	transcriptional antiterminator BglG
5.96	up	Cbei_2998	hypothetical protein
6	up	Cbei_2753	glycerol dehydrogenase
6.02	up	Cbei_0243	transcriptional anti-terminator BglG
6.02	up	Cbei_3973	MerR family transcriptional regulator
6.05	up	Cbei_0732	extracellular solute-binding protein
6.11	up	Cbei_4509	glycerol kinase
6.14	up	Cbei_3968	hypothetical protein
6.17	up	Cbei_4842	xylose isomerase domain-containing protein
6.17	up	Cbei_3468	flavodoxin
6.24	up	Cbei_2433	hypothetical protein
6.3	up	Cbei_0535	integral membrane sensor signal transduction histidine kinase
6.34	up	Cbei_1236	glycoside hydrolase
6.47	up	Cbei_2982	hypothetical protein
6.48	up	Cbei_4450	monosaccharide-transporting ATPase
6.52	up	Cbei_3486	methyl-accepting chemotaxis sensory transducer
6.59	up	Cbei_0804	methyl-accepting chemotaxis sensory transducer
6.6	up	Cbei_4511	MIP family channel protein
6.71	up	Cbei_1725	glycosyl transferase family protein

6.8	up	Cbei_4609	butyrate kinase
6.82	up	Cbei_0312	FAD linked oxidase domain-containing protein
6.86	up	Cbei_0644	hypothetical protein
6.96	up	Cbei_4680	extracellular solute-binding protein
6.97	up	Cbei_3122	inner-membrane translocator
6.99	up	Cbei_1728	hypothetical protein
7	up	Cbei_2997	hypothetical protein
7.02	up	Cbei_4727	cell wall binding repeat-containing protein
7.05	up	Cbei_4705	PTS system alpha-glucoside-specific transporter subunit IIBC
7.21	up	Cbei_2870	glycine/betaine ABC transporter substrate-binding protein
7.22	up	Cbei_0735	alpha amylase
7.24	up	Cbei_4554	HAD family hydrolase
7.37	up	Cbei_2384	xylulokinase
7.39	up	Cbei_2843	hypothetical protein
7.75	up	Cbei_0285	glycogen/starch/alpha-glucan phosphorylase
7.8	up	Cbei_4095	dihydroxy-acid dehydratase
7.89	up	Cbei_2488	hypothetical protein
7.95	up	Cbei_4555	sigma-54 factor interaction domain-containing protein
8.01	up	Cbei_4826	response regulator receiver modulated CheB methylesterase
8.05	up	Cbei_2489	hypothetical protein
8.12	up	Cbei_3287	---
8.29	up	Cbei_0965	PTS system sorbose-specific transporter subunit IIC
8.48	up	Cbei_1855	hypothetical protein
8.52	up	Cbei_3470	hypothetical protein
8.59	up	Cbei_4670	6-phospho-beta-glucosidase
8.62	up	Cbei_2741	PTS transporter subunit IIA-like nitrogen-regulatory protein PtsN
8.65	up	Cbei_3469	histidine kinase
8.73	up	Cbei_3325	ABC transporter
8.75	up	Cbei_2742	translaldolase
8.79	up	Cbei_3471	signal transduction protein
8.88	up	Cbei_0287	methyl-accepting chemotaxis sensory transducer
8.97	up	Cbei_4183	chemotaxis protein CheA
8.99	up	Cbei_0966	PTS system mannose/fructose/sorbose family transporter subunit IID
9.06	up	Cbei_3986	SCP-like extracellular protein
9.07	up	Cbei_1713	hemerythrin-like metal-binding protein
9.08	up	Cbei_0964	PTS system sorbose subfamily transporter subunit IIB
9.22	up	Cbei_3323	antibiotic transport-associated permease SpaG / MutG
9.27	up	Cbei_4832	methyl-accepting chemotaxis sensory transducer
9.3	up	Cbei_2160	multi-sensor hybrid histidine kinase
9.44	up	Cbei_2842	hypothetical protein
9.69	up	Cbei_4828	methyl-accepting chemotaxis sensory transducer

10.1	up	Cbei_1432	hypothetical protein
10.1	up	Cbei_3023	hypothetical protein
10.2	up	Cbei_4871	transketolase domain-containing protein
10.8	up	Cbei_0963	PTS system fructose subfamily transporter subunit IIA
11.1	up	Cbei_3324	lantibiotic permease spaE / mutE
11.1	up	Cbei_4829	chemotaxis protein CheA
11.2	up	Cbei_2039	sigma-54 dependent transcriptional regulator
11.4	up	Cbei_4546	xylose isomerase domain-containing protein
11.5	up	Cbei_2284	hydroxylamine reductase
11.8	up	Cbei_0242	PTS system mannitol-specific transporter subunit IIC
12.1	up	Cbei_4819	response regulator receiver protein
12.4	up	Cbei_0967	sugar isomerase (SIS)
12.5	up	Cbei_3130	GntR family transcriptional regulator
12.7	up	Cbei_0734	sugar ABC transporter permease
13	up	Cbei_3472	oxidoreductase FAD/NAD(P)-binding subunit
13.4	up	Cbei_4469	SCP-like extracellular protein
13.4	up	Cbei_0233	4-alpha-glucanotransferase
13.8	up	Cbei_2348	hypothetical protein
13.9	up	Cbei_2169	hypothetical protein
14	up	Cbei_0733	sugar ABC transporter permease
14.6	up	Cbei_4820	response regulator receiver sensor signal transduction histidine kinase
14.6	up	Cbei_1723	methyl-accepting chemotaxis sensory transducer
14.7	up	Cbei_4827	protein-glutamate O-methyltransferase
14.9	up	Cbei_3014	hydroxylamine reductase
15	up	Cbei_4824	response regulator receiver protein
15.9	up	Cbei_4818	hemerythrin-like metal-binding protein
16.5	up	Cbei_3356	methyl-accepting chemotaxis sensory transducer
16.9	up	Cbei_3625	methyl-accepting chemotaxis sensory transducer
17.2	up	Cbei_4549	myo-inositol catabolism IolB domain-containing protein
17.7	up	Cbei_3129	uridine phosphorylase
17.9	up	Cbei_4816	hemerythrin-like metal-binding protein
18.1	up	Cbei_4547	oxidoreductase domain-containing protein
18.1	up	Cbei_4550	ribokinase-like domain-containing protein
18.7	up	Cbei_2787	methyl-accepting chemotaxis sensory transducer
18.8	up	Cbei_4718	cell wall binding repeat-containing protein
19.4	up	Cbei_3288	metal dependent phosphohydrolase
21.2	up	Cbei_4817	hypothetical protein
21.4	up	Cbei_4821	methyl-accepting chemotaxis sensory transducer
21.8	up	Cbei_4551	fructose-bisphosphate aldolase
21.8	up	Cbei_2739	PTS system ascorbate-specific transporter subunit IIC
21.9	up	Cbei_4180	response regulator receiver modulated CheB methylesterase
23.7	up	Cbei_2740	"phosphotransferase system, lactose / cellobiose-specific IIB subunit"

23.8	up	Cbei_3128	cytidine deaminase
24.3	up	Cbei_4548	"thiamine pyrophosphate protein, central region"
24.7	up	Cbei_2166	hypothetical protein
24.8	up	Cbei_2850	methyl-accepting chemotaxis protein
25.4	up	Cbei_4823	methyl-accepting chemotaxis sensory transducer
25.5	up	Cbei_3127	phosphopentomutase
25.6	up	Cbei_3126	hypothetical protein
25.8	up	Cbei_0232	binding-protein-dependent transport system inner membrane protein
26	up	Cbei_2168	hypothetical protein
26	up	Cbei_2728	PAS/PAC sensor signal transduction histidine kinase
26.2	up	Cbei_4181	protein-glutamate O-methyltransferase
26.2	up	Cbei_2725	response regulator receiver sensor signal transduction histidine kinase
26.3	up	Cbei_4822	CheW protein
26.8	up	Cbei_4558	PTS system sorbose-specific transporter subunitIIC
28.9	up	Cbei_0782	formate/nitrite transporter
28.9	up	Cbei_2165	hemerythrin-like metal-binding protein
29.2	up	Cbei_2726	response regulator receiver protein
29.3	up	Cbei_3124	basic membrane lipoprotein
30	up	Cbei_2727	signal transduction protein
30.8	up	Cbei_3120	deoxyribose-phosphate aldolase
31.5	up	Cbei_0230	extracellular solute-binding protein
31.7	up	Cbei_4552	iron-containing alcohol dehydrogenase
33.4	up	Cbei_4182	methyl-accepting chemotaxis sensory transducer
34.6	up	Cbei_2167	hypothetical protein
38.8	up	Cbei_0231	binding-protein-dependent transport system inner membrane protein
40.5	up	Cbei_0784	anaerobic sulfite reductase subunit B
40.7	up	Cbei_4556	sugar isomerase (SIS)
42.3	up	Cbei_4560	PTS system fructose subfamily transporter subunit IIA
48.7	up	Cbei_0783	sulfite reductase subunit A
51.3	up	Cbei_3755	hypothetical protein
54.6	up	Cbei_4559	PTS system sorbose subfamily transporter subunit IIB
55.1	up	Cbei_4557	PTS system mannose/fructose/sorbose family transporter subunit IID
57.7	up	Cbei_3407	cell wall binding repeat-containing protein
75.2	up	Cbei_0785	sulfite reductase subunit C
82.1	up	Cbei_4184	CheW protein
88.8	up	Cbei_3125	purine nucleoside phosphorylase
5	down	Cbei_0140	50S ribosomal protein L11
5.05	down	Cbei_0146	30S ribosomal protein S12
5.06	down	Cbei_4027	CoA-substrate-specific enzyme activase
5.07	down	Cbei_1561	hypothetical protein
5.12	down	Cbei_0194	ribonucleotide-diphosphate reductase subunit alpha
5.14	down	Cbei_4159	LuxR family transcriptional regulator

5.15	down	Cbei_2451	RnfABCDGE type electron transport complex subunit G
5.16	down	Cbei_2675	coagulation factor 5/8 type domain-containing protein
5.19	down	Cbei_0246	glucosamine--fructose-6-phosphateaminotransferase
5.21	down	Cbei_1007	stationary phase survival protein SurE
5.25	down	Cbei_3115	hypothetical protein
5.26	down	Cbei_3944	hypothetical protein
5.3	down	Cbei_1604	pilus biogenesis protein
5.3	down	Cbei_2391	extracellular solute-binding protein
5.3	down	Cbei_2667	fatty acid desaturase
5.31	down	Cbei_4583	hypothetical protein
5.31	down	Cbei_1304	hypothetical protein
5.33	down	Cbei_2592	hypothetical protein
5.35	down	Cbei_2955	hypothetical protein
5.35	down	Cbei_1712	response regulator receiver protein
5.35	down	Cbei_0138	preprotein translocase subunit SecE
5.37	down	Cbei_1384	hypothetical protein
5.38	down	Cbei_4694	nucleotidyl transferase
5.39	down	Cbei_2697	hypothetical protein
5.4	down	Cbei_R0033	tRNA-Gly
5.41	down	Cbei_4778	hypothetical protein
5.43	down	Cbei_3013	hydrogenase (NiFe) small subunit HydA
5.44	down	Cbei_4137	hypothetical protein
5.48	down	Cbei_2685	abortive infection protein
5.5	down	Cbei_1968	aspartate/ornithine carbamoyl transferase family protein
5.51	down	Cbei_0509	hypothetical protein
5.6	down	Cbei_0601	hypothetical protein
5.63	down	Cbei_2594	hypothetical protein
5.63	down	Cbei_3034	response regulator receiver protein
5.66	down	Cbei_4157	radical SAM domain-containing protein
5.68	down	Cbei_4232	nucleoside-diphosphate kinase
5.77	down	Cbei_0635	5'-nucleotidase domain-containing protein
5.82	down	Cbei_0884	hypothetical protein
5.88	down	Cbei_0420	hypothetical protein
5.91	down	Cbei_2721	MerR family transcriptional regulator
5.91	down	Cbei_1119	"peptidase U4, sporulation factor SpoII GA"
5.92	down	Cbei_0592	VanW family protein
5.93	down	Cbei_3945	metallophosphoesterase
5.93	down	Cbei_3245	peptidoglycan binding domain-containing protein
5.96	down	Cbei_4623	cell wall binding repeat-containing protein
5.96	down	Cbei_1967	peptidase
5.98	down	Cbei_1559	uridine kinase
5.98	down	Cbei_0151	50S ribosomal protein L3
6	down	Cbei_1711	"peptidase S55, sporulation stage IV, protein B"
6.01	down	Cbei_2434	NLP/P60 protein
6.02	down	Cbei_1818	permease

6.02	down	Cbei_4198	FeoA family protein
6.07	down	Cbei_2410	flavodoxin
6.08	down	Cbei_0111	amidohydrolase
6.11	down	Cbei_3686	hypothetical protein
6.15	down	Cbei_3964	CHAP domain-containing protein
6.19	down	Cbei_1827	hypothetical protein
6.26	down	Cbei_2599	cysteine desulfurase
6.28	down	Cbei_0186	50S ribosomal protein L13
6.29	down	Cbei_5092	hypothetical protein
6.3	down	Cbei_0568	SpoVR family protein
6.39	down	Cbei_4613	hypothetical protein
6.4	down	Cbei_1882	hypothetical protein
6.44	down	Cbei_0508	50S ribosomal protein L21
6.44	down	Cbei_1390	methyl-accepting chemotaxis sensory transducer
6.45	down	Cbei_3043	hypothetical protein
6.53	down	Cbei_4581	---
6.53	down	Cbei_0500	hypothetical protein
6.58	down	Cbei_2774	cell wall binding repeat-containing protein
6.58	down	Cbei_2448	sigma E positive regulator RseC/MucC
6.61	down	Cbei_0427	hypothetical protein
6.63	down	Cbei_4614	coat F domain-containing protein
6.65	down	Cbei_1087	diguanylate cyclase
6.66	down	Cbei_1848	FeS assembly ATPase SufC
6.68	down	Cbei_5053	BadM/Rrf2 family transcriptional regulator
6.71	down	Cbei_3113	hypothetical protein
6.85	down	Cbei_1211	aspartate kinase I
7.08	down	Cbei_3683	cell wall-associated hydrolase-like protein
7.13	down	Cbei_2606	hypothetical protein
7.15	down	Cbei_2439	cytochrome b5
7.31	down	Cbei_2578	"dTDP-glucose 4,6-dehydratase"
7.32	down	Cbei_3981	extracellular solute-binding protein
7.39	down	Cbei_4106	ErfK family protein
7.41	down	Cbei_0850	spore coat protein CotS
7.43	down	Cbei_2260	hypothetical protein
7.44	down	Cbei_1535	membrane protein
7.52	down	Cbei_0391	peptidoglycan-binding LysM
7.56	down	Cbei_1988	molybdopterin binding domain-containing protein
7.57	down	Cbei_1969	carbamate kinase
7.57	down	Cbei_5029	DEAD/DEAH box helicase
7.62	down	Cbei_0426	hypothetical protein
7.73	down	Cbei_1973	selenate reductase subunit YgfK
7.76	down	Cbei_2344	hypothetical protein
7.83	down	Cbei_4612	coat F domain-containing protein
7.84	down	Cbei_3167	hypothetical protein
7.89	down	Cbei_1282	di-trans-poly-cis-decaprenylcis transferase
8.01	down	Cbei_3250	"small acid-soluble spore protein, alpha/betatype"
8.02	down	Cbei_0051	nucleoside recognition domain-containing protein
8.14	down	Cbei_4221	cell wall binding repeat-containing protein
8.28	down	Cbei_2069	manganese containing catalase

8.28	down	Cbei_0093	YabP family protein
8.33	down	Cbei_2954	cupin
8.37	down	Cbei_0088	AbrB family transcriptional regulator
8.4	down	Cbei_2598	amine oxidase
8.41	down	Cbei_2390	GntR family transcriptional regulator
8.53	down	Cbei_4365	hypothetical protein
8.57	down	Cbei_1136	"sporulation stage IV, protein A"
8.72	down	Cbei_0094	spore cortex biosynthesis protein YabQ
8.73	down	Cbei_0812	anti-sigma-factor antagonist
8.74	down	Cbei_3275	"small acid-soluble spore protein, alpha/betatype"
8.8	down	Cbei_2827	---
9.01	down	Cbei_4243	ATPase P
9.02	down	Cbei_1945	signal-transduction protein
9.04	down	Cbei_3170	hypothetical protein
9.04	down	Cbei_4068	hypothetical protein
9.08	down	Cbei_4023	cell wall hydrolase/autolysin
9.08	down	Cbei_3923	integral membrane sensor signal transduction histidine kinase
9.17	down	Cbei_1987	LysR family transcriptional regulator
9.3	down	Cbei_3684	ATPase AAA
9.32	down	Cbei_3761	transport system permease
9.39	down	Cbei_1930	hypothetical protein
9.43	down	Cbei_0813	anti-sigma F factor
9.44	down	Cbei_3168	phosphoglycerate mutase
9.47	down	Cbei_3685	hypothetical protein
9.47	down	Cbei_0574	hypothetical protein
9.54	down	Cbei_0689	hypothetical protein
9.59	down	Cbei_4329	hypothetical protein
9.59	down	Cbei_3572	ApbE family lipoprotein
9.75	down	Cbei_0488	hypothetical protein
9.81	down	Cbei_1120	sporulation sigma factor SigE
9.82	down	Cbei_0814	sporulation sigma factor SigF
9.92	down	Cbei_3380	cell wall binding repeat-containing protein
10	down	Cbei_0691	acyl-ACP thioesterase
10	down	Cbei_3268	"holin, phage phi LC3"
10.1	down	Cbei_3571	FMN-binding domain-containing protein
10.2	down	Cbei_2581	hypothetical protein
10.2	down	Cbei_0571	hypothetical protein
10.3	down	Cbei_4022	glycoside hydrolase family protein
10.5	down	Cbei_3171	accessory gene regulator B
10.5	down	Cbei_0688	beta-lactamase domain-containing protein
10.7	down	Cbei_2595	hypothetical protein
10.7	down	Cbei_4878	single-strand binding protein
10.8	down	Cbei_3834	3-oxoacid CoA-transferase subunit B
10.9	down	Cbei_1863	hypothetical protein
10.9	down	Cbei_0566	serine protein kinase PrkA
11.2	down	Cbei_1816	"cobalamin synthesis protein, P47K"
11.4	down	Cbei_4700	hypothetical protein
11.4	down	Cbei_0150	30S ribosomal protein S10

11.5	down	Cbei_2583	hypothetical protein
11.7	down	Cbei_0823	stage II sporulation P family protein
12.5	down	Cbei_1826	RNA polymerase sigma factor SigI
12.7	down	Cbei_2828	glycoside hydrolase
12.7	down	Cbei_1789	polysaccharide deacetylase
12.8	down	Cbei_3763	periplasmic binding protein
12.9	down	Cbei_0282	hypothetical protein
13	down	Cbei_1693	stage III sporulation protein SpoAB
13	down	Cbei_4079	hypothetical protein
13.1	down	Cbei_0757	ErfK family protein
13.1	down	Cbei_1558	hypothetical protein
13.2	down	Cbei_3169	multi-sensor signal transduction histidine kinase
13.2	down	Cbei_0586	hypothetical protein
13.6	down	Cbei_2600	hypothetical protein
13.6	down	Cbei_1698	"sporulation stage III, protein AG"
13.8	down	Cbei_4584	ABC transporter
13.8	down	Cbei_0682	hypothetical protein
14	down	Cbei_3256	hypothetical protein
14.3	down	Cbei_4337	thiamine pyrophosphate binding domain-containing protein
14.7	down	Cbei_0060	hypothetical protein
15	down	Cbei_0283	ABC transporter
15	down	Cbei_3688	hypothetical protein
15.3	down	Cbei_0061	hypothetical protein
15.4	down	Cbei_0354	hypothetical protein
15.6	down	Cbei_0281	hypothetical protein
15.9	down	Cbei_1061	hypothetical protein
16	down	Cbei_1696	"sporulation stage III, protein AE"
16.1	down	Cbei_0358	hypothetical protein
16.2	down	Cbei_3326	hypothetical protein
16.7	down	Cbei_2826	carbohydrate-binding family V/XII protein
16.8	down	Cbei_2830	glycoside hydrolase
17.1	down	Cbei_3495	beta-lactamase domain-containing protein
17.4	down	Cbei_0690	radical SAM domain-containing protein
17.6	down	Cbei_3197	glycosyl transferase family protein
17.9	down	Cbei_0015	ErfK/YbiS/YcfS/YnhG family protein
17.9	down	Cbei_0591	glycoside hydrolase
18.5	down	Cbei_0045	hypothetical protein
18.7	down	Cbei_0284	hypothetical protein
19	down	Cbei_4690	membrane spanning protein
19	down	Cbei_2831	carbohydrate-binding family V/XII protein
19.1	down	Cbei_0677	EmrB/QacA family drug resistance transporter
19.7	down	Cbei_0280	hypothetical protein
19.9	down	Cbei_0684	AMP-dependent synthetase and ligase
20.3	down	Cbei_0422	sporulation stage II protein D
20.8	down	Cbei_3833	3-oxoacid CoA-transferase subunit A
21	down	Cbei_0097	"sporulation stage II, protein E"
21.7	down	Cbei_1692	"sporulation stage III, protein AA"
22	down	Cbei_3207	cell wall binding repeat-containing protein

22.8	down	Cbei_0567	hypothetical protein
22.9	down	Cbei_4689	hypothetical protein
23	down	Cbei_3835	acetoacetate decarboxylase
23.2	down	Cbei_1121	sporulation sigma factor SigG
23.5	down	Cbei_3330	type I phosphodiesterase/nucleotide pyrophosphatase
24.2	down	Cbei_4336	class V aminotransferase
24.8	down	Cbei_1888	hypothetical protein
24.9	down	Cbei_2261	lytic transglycosylase
25	down	Cbei_0687	4'-phosphopantetheinyl transferase
25.3	down	Cbei_0686	AMP-dependent synthetase and ligase
25.5	down	Cbei_3836	"peptidase C1A, papain"
25.6	down	Cbei_3687	hypothetical protein
26.3	down	Cbei_1695	"sporulation stage III, protein AD"
26.4	down	Cbei_3832	aldehyde dehydrogenase
26.4	down	Cbei_0044	hypothetical protein
26.8	down	Cbei_1212	"peptidase S14, ClpP"
26.8	down	Cbei_3048	---
27.2	down	Cbei_1699	stage III sporulation protein AH
28.5	down	Cbei_5022	hypothetical protein
28.7	down	Cbei_1122	sporulation protein YlmC/YmxH
29.3	down	Cbei_0199	cell wall hydrolase SleB
29.6	down	Cbei_2155	hypothetical protein
30.1	down	Cbei_3049	ABC transporter
30.2	down	Cbei_3332	aliphatic sulfonate ABC transporter periplasmicprotein
30.4	down	Cbei_4339	nucleotidyl transferase
30.7	down	Cbei_4338	cytidyltransferase-like protein
33.3	down	Cbei_0685	alcohol dehydrogenase
33.3	down	Cbei_0424	"sporulation stage III, transcriptional regulatorSpoIIID"
36.2	down	Cbei_2653	3-oxoacid CoA-transferase subunit B
36.3	down	Cbei_0683	radical SAM domain-containing protein
40.3	down	Cbei_0674	NAD-dependent aldehyde dehydrogenase-likeprotein
40.6	down	Cbei_0680	O-methyltransferase family protein
40.9	down	Cbei_4587	hypothetical protein
41.3	down	Cbei_0681	thioesterase
42.4	down	Cbei_0569	rubrerythrin
45.6	down	Cbei_0679	ABC transporter
46.6	down	Cbei_0570	hypothetical protein
48.9	down	Cbei_0657	type 11 methyltransferase
51.9	down	Cbei_1963	phage integrase family protein
55.3	down	Cbei_0387	spore coat protein CotS
65.2	down	Cbei_0673	---
67.8	down	Cbei_0423	peptidase M23B
75.4	down	Cbei_0675	coenzyme F390 synthetase-like protein
84.5	down	Cbei_1962	"sporulation stage II, protein M"

Table S5a Fold change of sugar transporter genes in DG-8052 vs WT-8052 as determined by microarray analysis

Gene symbol	Gene product	Fold Change (Acidogenic phase)	Fold Change (Solventogenic phase)
**Phosphostransferase system (PTS)**			
Cbei_0222	PTS system Galactitol-specific IIC component	+1.84	+2.34
Cbei_0242	PTS system mannitol-specific transporter subunitIIC	+10	+11.8
Cbei_0244	phosphoenolpyruvate-dependent sugar phosphotransferase system, EIIA 2	+5.87	+3.96
Cbei_0339	PTS system glucitol/sorbitol-specific IIA component	+3.48	+3.86
Cbei_0543	PTS system Galactitol-specific IIC component	+1.25	+2.06
Cbei_0758	phosphotransferase system, lactose/cellobiose-specific IIB subunit	+3.61	+2.95
Cbei_0950	phosphotransferase system, EIIC	+1.90	+3.07
Cbei_0951	phosphotransferase system, lactose/cellobiose-specific IIB subunit	+2.45	+2.96
Cbei_0958	PTS system mannose/fructose/sorbose family IID component	+2.09	+2.45
Cbei_0963	PTS system fructose subfamily IIA component	+9.29	+10.8
Cbei_0965	phosphotransferase system PTS, sorbose-specific IIC subunit	+5.61	+8.29
Cbei_0966	PTS system mannose/fructose/sorbose familyIID component	+8.22	+8.99
Cbei_1918	phosphotransferase system PTS, EIIB protein; phosphotransferase system, EIIC	+3.04	+3.84
Cbei_2708	phosphotransferase system, lactose/cellobiose-specific IIB subunit	+2.58	+2.87
Cbei_2740	phosphotransferase system, lactose/cellobiose-specific IIB subunit	+34.9	+23.7
Cbei_2741	phosphoenolpyruvate-dependent sugar phosphotransferase system, EIIA 2	+62.4	+8.62
Cbei_2833	sugar-specific permease, EIIA 1 domain; phosphotransferase system PTS, EIIB protein; phosphotransferase system, EIIC	+2.71	+2.09
Cbei_3871	PTS system mannose/fructose/sorbose family IID component	+1.28	+2.33
Cbei_4557	PTS system mannose/fructose/sorbose family IID component	+42.3	+55.1
Cbei_4558	phosphotransferase system PTS, sorbose-specific IIC subunit	+22.1	+26.8
Cbei_4559	PTS system sorbose subfamily IIB component	+38.9	+54.6
Cbei_4639	phosphotransferase system, lactose/cellobiose-specific IIB subunit	+2.96	+2.89

Cbei_4684	phosphotransferase system, lactose/cellobiose-specific IIB subunit	+3.65	+3.18
Cbei_4685	phosphotransferase system PTS, lactose/cellobiose-specific IIA subunit	+3.56	+7.05
Cbei_4705	phosphotransferase system PTS, EIIB protein; phosphotransferase system, EIIC	+4.55	+7.05
Cbei_4838	sugar-specific permease, EIIA 1 domain; phosphotransferase system PTS, EIIB protein; phosphotransferase system, EIIC	+2.0	+3.14
Cbei_0336	PTS system protein II sorbitol-specific factor	+3.84	+4.95
**ABC transporter**			
Cbei_0283	ABC transporter	-12.1	-14.96
Cbei_1380	periplasmic binding protein/LacItranscriptional regulator	-2.51	-2.13
Cbei_1762	Extracellular ligand-binding receptor	-3.11	-1.39
Cbei_1763	inner-membrane translocator	-3.4	-1.78
Cbei_1764	inner-membrane translocator	-2.73	-1.25
Cbei_1767	Extracellular ligand-binding receptor	-2.13	-1.11
Cbei_2668	ABC transported MDR-type, permease component	-2.68	-2.09
Cbei_3332	Substrate-binding region of ABC-type glycine betaine transport system	-4.23	-30.24
Cbei_3760	ABC transporter	-3.69	-4.35
Cbei_3761	transport system permease	-4.2	-9.32
Cbei_3763	periplasmic binding protein	-3.36	-12.79
Cbei_3826	ABC transporter	-2.09	-2.32
Cbei_4190	ABC transporter; Transport-associated OB domain protein	-2.27	+1.28
Cbei_4191	binding-protein-dependent transport systems inner membrane component	-2.19	+1.27
Cbei_4192	binding-protein-dependent transport systems inner membrane component	-3.19	-1.28
Cbei_4194	extracellular solute-binding protein, family 1	-3.84	-1.69
Cbei_4335	ABC transporter	-2.26	-4.09
Cbei_4928	extracellular solute-binding protein, family5	-2.37	-3.71
Cbei_4929	ABC transporter; Oligopeptide/dipeptide ABC transporter C-terminal domain protein	-2.19	-2.45
Cbei_4930	ABC transporter; Oligopeptide / dipeptide ABC transporter C-terminal domain protein	-2.38	-3.06
Cbei_4931	binding-protein-dependent transport systems inner membrane component	-2.87	-2.96
Cbei_4932	binding-protein-dependent transport systems inner membrane component	-2.31	-2.74

Cbei_5042	Extracellular ligand-binding receptor	-6.57	-4.49
Cbei_5043	inner-membrane translocator	-2.52	-1.28
Cbei_5044	inner-membrane translocator	-4.72	-1.68
Cbei_5045	ABC transporter	-3.39	-1.58
Cbei_5046	ABC transporter	-3.75	-1.50

Table S5b Fold change of sigma-54 & sugar metabolism genes in DG-8052 vs 8052 as determined by microarray analysis

Gene symbol	Gene product	Fold Change	
		Acidogenic phase	Solventogenic phase
**Sigma factor**			
Cbei_0595	RNA polymerase factor sigma-54	+5.75	+4.08
**Pentose phosphate pathway**			
Cbei_0227	sugar-phosphate isomerase, RpiB/LacA/LacB family; ribose 5-phosphate isomerase B	+3.16	+3.61
Cbei_0338	Transaldolase	+3.08	+3.22
Cbei_0462	2-dehydro-3-deoxyphosphogluconatealdolase/4-hydroxy-2-oxoglutarate aldolase	+2.02	+2.27
Cbei_0584	catalyzes the formation of D-fructose1,6-bisphosphate from D-fructose 6-phosphate in glycolysis	+3.18	+2.71
Cbei_2742	similar to novel fructose-6-phosphate aldolase from *Escherichia coli*; enzyme from *Methanocaldococcus janaschii* shows transaldolase activity	+39.2	+8.75
Cbei_3120	catalyzes the formation of D-glyceraldehyde3-phosphate and acetaldehyde from2-deoxy-D-ribose-5-phosphate	+19.4	+30.8
Cbei_4551	catalyzes the formation of glycerone phosphate and glyceraldehyde 3-phosphate from fructose 1,6,bisphosphate	+15.2	+21.8
Cbei_4648	PfkB domain protein	+3.04	+2.57
Cbei_4871	Transketolase domain protein	+13.1	+10.2
Cbei_3039	catalyzes the formation of glycerone phosphate and D-glyceraldehyde 3-phosphate from D-fructose1,6-bisphosphate in glycolysis	+3.11	+1.57
Cbei_3127	catalyzes the transfer of phosphate between the C1and C5 carbons of pentose	+26.6	+25.5
**Fructose and mannose metabolism**			
Cbei_0242	PTS system, mannitol-specific IIC subunit	+10	+11.8
Cbei_0244	phosphoenolpyruvate-dependent sugar phosphotransferase system, EIIA 2	+5.87	+3.96

Cbei_0245	Mannitol dehydrogenase, C-terminal domain; Mannitol dehydrogenase rossman, N-terminal domain	+5.37	+4.65
Cbei_0339	PTS system, glucitol/sorbitol-specific IIA subunit	+3.48	+3.86
Cbei_0445	rhamnulokinase	+3.1	+2.5
Cbei_0584	catalyzes the formation of D-fructose1,6-bisphosphate from D-fructose 6-phosphate in glycolysis	+3.18	+2.71
Cbei_0958	PTS system mannose/fructose/sorbose familyIID component	+2.09	+2.45
Cbei_0963	"PFAM: PTS system fructose subfamily IIA component	+9.29	+10.8
Cbei_0965	phosphotransferase system PTS, sorbose-specific IIC subunit	+5.61	+8.29
Cbei_0966	PTS system mannose/fructose/sorbose familyIID component	+8.22	+8.99
Cbei_4551	catalyzes the formation of glycerone phosphate and glyceraldehyde 3-phosphate from fructose 1,6,bisphosphate	+15.2	+2.33
Cbei_4557	PTS system mannose/fructose/sorbose familyIID component	+42.3	+21.8
Cbei_4558	phosphotransferase system PTS, sorbose-specific IIC subunit	+22.1	+55.1
Cbei_4559	PTS system sorbose subfamily IIB component	+38.9	+54.6
**Starch and sucrose metabolism**			
Cbei_0233	amylomaltase; acts to release glucose frommaltodextrins	+9.45	+13.4
Cbei_0864	amylomaltase; acts to release glucose frommaltodextrins	+2.11	+3.03
Cbei_1443	glycoside hydrolase, family 31	+3.02	+1.47
Cbei_1918	phosphotransferase system PTS, EIIB protein; phosphotransferase system, EIIC	+3.04	+3.84
Cbei_3773	sucrose-6-phosphate hydrolase	+3.31	+3.75
Cbei_4618	UTP-glucose-1-phosphateuridylyltransferase	+4.59	+2.91
Cbei_4909	1,4-alpha-glucan branching enzyme	+2.01	+2.5

Cbei_4984	glycoside hydrolase, family 4	+3.97	+2.57
**Galactose metabolism**			
Cbei_0584	catalyzes the formation of D-fructose1,6-bisphosphate from D-fructose 6-phosphate in glycolysis	+3.18	+2.71
Cbei_1236	glycoside hydrolase, family 42, domain 5,loop region; glycoside hydrolase family 2, immunoglobulin domain protein beta-sandwich; glycoside hydrolase family2, TIM barrel; glycoside hydrolase family 2, sugar binding	+6.16	+6.34
Cbei_1443	glycoside hydrolase, family 31	+3.02	+1.47
Cbei_3773	sucrose-6-phosphate hydrolase	+3.31	+3.75
Cbei_4359	glycoside hydrolase, family 4	+2.15	+2.03
Cbei_4618	UTP-glucose-1-phosphateuridylyltransferase	+4.59	+2.91
**Glycolysis/gluconeogenesis**			
Cbei_0584	catalyzes the formation of D-fructose1,6-bisphosphate from D-fructose 6-phosphate in glycolysis	+3.18	+2.71
Cbei_1719	possible phosphoglycerate mutase	+2.03	+2.14
Cbei_3271	phospho-beta-glucosidase	+1.93	+2.2
Cbei_3984	6-phospho-beta-glucosidase	+1.45	+2.41
Cbei_4036	6-phospho-beta-glucosidase	+1.82	+2.34
Cbei_4465	sus:Acid_1458 aldose 1-epimerase	+5.58	+4.73
Cbei_4551	catalyzes the formation of glycerone phosphate and glyceraldehyde 3-phosphate from fructose 1,6,bisphosphate	+3.28	+21.8
Cbei_4669	beta-glucosidase	+2.70	+4.03
Cbei_4670	glycosyl hydrolase, family 4	+11.46	+8.59
Cbei_4705	maltose-specific enzyme IICB component	+4.55	+7.05
Cbei_5017	PTS glucose-specific enzyme IIA component	+1.75	+2.38
**Inositol phosphate metabolism**			
Cbei_4547	myo-inositol 2-dehydrogenase	+9.34	+18.10
Cbei_4548	myo-inositol catabolism protein	+15.05	+24.30

Cbei_4549	myo-inositol catabolism protein iol B	+12.08	+17.20

Table S5c Fold change of sigma-70 family and sporulation genes in DG-8052 vs WT-8052 as determined by microarray analysis

Gene symbol	Gene product	Fold Change	
		Acidogenic phase	Solventogenic phase
Cbei_0812	anti-sigma F factor antagonist	-4.25	-8.73
Cbei_0813	Anti-sigma F factor	-3.57	-9.43
Cbei_0814	sporulation sigma factor SigF	-3.64	-9.82
Cbei_1119	sporulation factor SpoIIGA	-1.20	-5.91
Cbei_1120	sigma-29; sporulation sigma factor SigE	+1.99	-9.81
Cbei_1121	sporulation sigma factor SigG	+1.01	-22.22
Cbei_0422	stage II sporulation protein D	-1.74	-20.31
Cbei_1712	Spo0A protein	-3.16	-5.35
Cbei_0135	RNA polymerase factor sigma-70	-2.06	-1.77

Table S5d Fold change of *spo0A* and chemotasis genes in DG-8052 vs WT-8052 as determined by microarray analysis

Gene symbol	Gene product	Fold Change	
		Acidogenic phase	Solventogenic phase
Cbei_1712	Spo0A protein	-3.16	-5.35
**Chemotasis**			
Cbei_0287	histidine kinase, HAMP region domain protein; Cache domain protein; chemotaxis sensory	+4.4	+8.88
Cbei_0804	histidine kinase, HAMP region domain protein; chemotaxis sensory transducer	+5.16	+6.59
Cbei_2787	histidine kinase, HAMP region domain protein; chemotaxis sensory transducer	+16.6	+18.7
Cbei_3045	histidine kinase, HAMP region domain protein; chemotaxis sensory transducer	+2.01	+2.42
Cbei_3320	histidine kinase, HAMP region domain protein; Cache domain protein; chemotaxis sensory	+4.55	+5.23
Cbei_3356	histidine kinase, HAMP region domain protein; chemotaxis sensory transducer	+10.2	+16.5
Cbei_3625	histidine kinase, HAMP region domain protein; Cache domain protein; chemotaxis sensory transducer	+13.6	+16.9
Cbei_3671	histidine kinase, HAMP region domain protein; chemotaxis sensory transducer	+4.39	+5.16
Cbei_4015	response regulator receiver	+1.93	+2.59
Cbei_4017	histidine kinase, HAMP region domain protein; chemotaxis sensory transducer	+1.84	+3.5
Cbei_4018	CheW domain protein	+1.47	+2.21
Cbei_4180	CheB methylesterase; response regulator receiver	+16.4	+21.9
Cbei_4181	MCP methyltransferase, CheR-type	+16.9	+26.2
Cbei_4182	chemotaxis sensory transducer	+36.9	+33.4
Cbei_4183	CheW domain protein; ATP-binding region, ATPase domain protein domain protein; Signal transducing histidine kinase, homodimeric; Hpt domain protein; P2response regulator binding domain protein	+20.9	+8.97
Cbei_4184	CheW domain protein	+59.4	+82.1
Cbei_4463	periplasmic binding protein/LacI transcriptional regulator	+1.98	+2.35
Cbei_4819	response regulator receiver	+11.1	+12.1

Cbei_4821	histidine kinase, HAMP region domain protein; chemotaxis sensory transducer	+21.4	+21.4
Cbei_4822	CheW domain protein	+20.4	+26.3
Cbei_4826	CheB methylesterase; response regulator receiver	+4.11	+8.01
Cbei_4827	MCP methyltransferase, CheR-type	+12.5	+14.7
Cbei_4828	histidine kinase, HAMP region domain protein; chemotaxis sensory transducer	+10.9	+9.69
Cbei_4829	CheW domain protein; ATP-binding region, ATPase domain protein domain protein; Signal transducing histidine kinase, homodimeric; Hpt domain protein; P2response regulator binding domain protein	+11.1	+11.1

Table S5e Fold change of signal transductions genes in DG-8052 vs WT-8052 as determined by microarray analysis

Gene symbol	Gene product	Fold Change	
		Acidogenic phase	Solventogenic phase
**two-component signal transduction system**			
Cbei_0411	acetyl-CoA acetyltransferase	+1.56	+2.02
Cbei_0534	response regulator receiver; transcriptional regulator domain	+2.29	+3.12
Cbei_0589	response regulator receiver; transcriptional regulator domain	+3.89	+3.40
Cbei_0768	histidine kinase, HAMP region domain protein; histidine kinase internal region	+2.21	-3.15
Cbei_1125	response regulator receiver; transcriptional regulator domain protein	+2.82	+2.35
Cbei_1127	extracellular solute-binding protein, family 1	+2.78	+2.92
Cbei_2249	response regulator receiver; transcriptional regulator domain protein	+2.17	+2.21
Cbei_2378	ATP-binding region, ATPase domain protein domain protein; histidine kinase, HAMP region domain protein; histidine kinase internal region	+4.94	+2.76
Cbei_2484	response regulator receiver; transcriptional regulator domain protein	+3.99	+4.1
Cbei_2725	response regulator receiver; ATP-binding region, ATPase domain protein domain protein;	+17.75	+26.17
Cbei_2726	response regulator receiver	+28.71	+29.2
Cbei_2727	response regulator receiver; Metal-dependent hydrolase HDOD	+25.69	+29.97
Cbei_2951	response regulator receiver; transcriptional regulator domain	+4.82	+4.26
Cbei_3322	response regulator receiver; transcriptional regulator domain protein	+4.88	+5.60
Cbei_3471	response regulator receiver; Metal-dependent hydrolase HDOD	+7.38	+8.79
Cbei_3663	response regulator receiver; transcriptional regulator domain protein	+2.33	+2.60
Cbei_4004	response regulator receiver	+3.58	+3.48
Cbei_4005	ATP-binding region, ATPase domain protein domain protein; PAS fold domain protein	+2.70	+2.55
Cbei_4015	response regulator receiver	+1.93	+2.59
Cbei_4018	Che W domain protein	+1.47	+2.59
Cbei_4051	helix-turn-helix- domain containing protein, AraC type; response regulator receiver	+3.96	+3.49

Cbei_4052	ATP-binding region, ATPase domain protein domain protein; histidine kinase internal region	+3.07	+2.93
Cbei_4103	response regulator receiver; transcriptional regulator domain	+4.45	+2.29
Cbei_4180	CheB methylesterase; response regulator receiver	+16.42	+21.92
Cbei_4181	MCP methyltransferase, CheR-type	+16.88	+26.2
Cbei_4183	CheW domain protein; ATP-binding region, ATPase domain protein domain protein; Signal transducing histidine kinase, homodimeric; Hpt domain protein; P2 response regulator binding domain protein	+20.88	+8.97
Cbei_4184	CheW domain protein	+59.42	+82.1
Cbei_4431	helix-turn-helix- domain containing protein, AraC type; response regulator receiver	+2.79	+2.80
Cbei_4688	GGDEF domain containing protein; histidine kinase, HAMP region domain protein; PAS fold-3 domain protein; PAS fold domain protein; SMART: PAC repeat-containing protein	+12.37	+2.38
Cbei_4808	GGDEF domain containing protein; metal-dependent phosphohydrolase, HD sub domain; PAS fold-4 domain protein; PAS fold domain protein; SMART: PAS domain containing protein; PAC repeat-containing protein; metal-dependent phosphohydrolase, HD region	+2.88	+3.25
Cbei_4813	response regulator receiver	+4.14	+3.68
Cbei_4815	hypothetical protein	+3.12	+5.20
Cbei_4819	response regulator receiver	+16.06	+12.1
Cbei_4822	CheW domain protein	+20.41	+26.3
Cbei_4824	response regulator receiver	+17.91	+15.00
Cbei_4826	CheB methylesterase; response regulator receiver	+4.11	+8.01
Cbei_4827	MCP methyltransferase, CheR-type	+12.50	+14.7
Cbei_4829	CheW domain protein; ATP-binding region, ATPase domain protein domain protein; Signal transducing histidine kinase, homodimeric; Hpt domain protein; P2response regulator binding domain protein	+11.06	+11.1
Cbei_5033	nitrogen regulatory protein P-II	+1.45	+2.1
**sensor protein**			
Cbei_4820	response regulator receiver; ATP-binding region, ATPase domain protein domain protein; histidinekinase A domain protein domain protein	+15.25	+14.58

Cbei_2160	response regulator receiver; ATP-binding region, ATPase domain protein domain protein; histidine kinase, HAMP region domain protein; histidine kinase A domain protein domain protein; Hpt domain protein	+6.39	+9.30
Cbei_4102	ATP-binding region, ATPase domain protein domain protein; histidine kinase, HAMP region domain protein; histidine kinase A domain protein domain protein	+2.4	+2.37
Cbei_2485	ATP-binding region, ATPase domain protein domain protein; histidine kinase, HAMP region domain protein; histidine kinase A domain protein domain protein	+3.58	+1.22
Cbei_2950	ATP-binding region, ATPase domain protein domain protein; histidine kinase, HAMP region domain protein; histidine kinase A domain protein domain protein	+4.21	+3.58
Cbei_3321	ATP-binding region, ATPase domain protein domain protein; histidine kinase, HAMP region domain protein; histidine kinase A domain protein domain protein	+3.78	+3.60
Cbei_3665	ATP-binding region, ATPase domain protein domain protein; histidine kinase A domain protein domain protein	+2.12	+2.13
Cbei_4814	response regulator receiver; ATP-binding region, ATPase domain protein domain protein; histidine kinase A domain protein domain protein	+3.18	+4.19
Cbei_0535	ATP-binding region, ATPase domain protein domain protein; histidine kinase A domain protein domain protein	+4.26	+6.30
Cbei_3662	ATP-binding region, ATPase domain protein domain protein; histidine kinase, HAMP region domain protein; histidine kinase A domain protein domain protein	+2.45	+2.65
Cbei_2250	ATP-binding region, ATPase domain protein domain protein; histidine kinase, HAMP region domain protein; histidine kinase A domain protein domain protein	+2.64	+3.08

Table S5f Fold change of solvent production genes in DG-8052 vs WT-805 as determined by microarray analysis

Gene Symbol	Gene Product	Fold Change	
		Acidogenic phase	Solventogenic phase
Cbei_3516<br>Cbei_2063	pyruvate synthase	+1.26<br>+1.98	+1.25<br>+1.98
Cbei_1165	acetate kinase	+2.93	+3.26
Cbei_1164	phosphotransacetylase	+2.75	-1.05
Cbei_1953<br>Cbei_3832<br>Cbei_0674<br>Cbei_2518	aldehyde dehydrogenase	-3.34<br>-3.11<br>-50.64<br>-2.62	-2.49<br>-26.35<br>-40.26<br>-3.44
Cbei_1932<br>Cbei_4552<br>Cbei_0223<br>Cbei_0685	alcohol dehydrogenase	+3.73<br>+19.97<br>+2.47<br>-24.87	+5.03<br>+31.65<br>+2.77<br>-33.26
Cbei_3630<br>Cbei_0411	thiolase	+1.72<br>+1.56	+1.95<br>+2.02
Cbei_3835	acetoacetate decarboxylase	-23.22	-22.99
Cbei_3819<br>Cbei_2040<br>Cbei_3278	acetoacetyl-CoA transferase subunit A	+2.95<br>+2.07<br>-1.09	+5.6<br>+1.9<br>+2.15
Cbei_3833	butyrate-acetoacetate CoA-transferase subunit A	-14.34	-20.78
Cbei_3834	butyrate-acetoacetate CoA-transferase subunit B	-9.68	-10.83
Cbei_0325<br>Cbei_0324<br>Cbei_2037	3-hydroxybutyryl-CoA dehydrogenase	+1.66<br>-1.08<br>-1.14	+1.23<br>-1.42<br>-1.31
Cbei_2230	Enoyl-CoA hydratase	+1.82	+3.21
Cbei_2883<br>Cbei_3820	butyryl-CoA dehydrogenase	+3.47<br>+2.27	+2.69<br>+2.88
Cbei_4609	butyrate kinase	+4.92	+6.8
Cbei_4899	phosphotrans butyrylase	-2.17	-2.43
