# Supplementary material for: Sigma Factor Regulated Cellular Response in a Non-solvent Producing Clostridium beijerinckii Degenerated Strain: A Comparative Transcriptome Analysis
Source: Front Microbiol. 2017 Jan 30;8:23. doi: 10.3389/fmicb.2017.00023 (PMC5276810; doi:10.3389/fmicb.2017.00023)
Supplement: Supplementary file 2 [file Image_1.PDF]

Fig.S1 q-RT-PCR analysis results to show the mRNA levels changes of the related genes (1, heat shock protein Hsp20, Cbei_4123; 2, acetyl-CoA acetyltransferase, Cbei_0411; 3, electron transfer flavoprotein subunitalpha-like protein, Cbei_0311; 4, deoxyribose-phosphate aldolase, Cbei_3120; 5, Permease, Cbei_0441; 6, inosine 5'-monophosphate dehydrogenase, Cbei_0331; 7, response regulator receiver protein, Cbei_4824; 8, phosphotransferase system,lactose/cellobiose-specific IIB subunit, Cbei_2740; 9, transketolase domain-containing protein, Cbei_4871; 10, methyl-accepting chemotaxis sensory transducer, Cbei_3356) at 12h fermentation time, (11, acetoacetate decarboxylase , Cbei_3835; 12, hypothetical protein , Cbei_1930; 13, EmrB/QacA family drug resistance transporter , Cbei_0677; 14, carbohydrate-binding family V/XII protein , Cbei_2826; 15, hypothetical protein , Cbei_2600; 16, stage V sporulation protein E , Cbei_1583; 17, hypothetical protein, Cbei_1079 ; 18, hypothetical protein, Cbei_0284; 19, aldehyde dehydrogenase, Cbei_3832; 20, lytic transglycosylase, Cbei_2261) at 24h fermentation time in DG-8052 cells VS WT-8052 cells.

Fig.S1

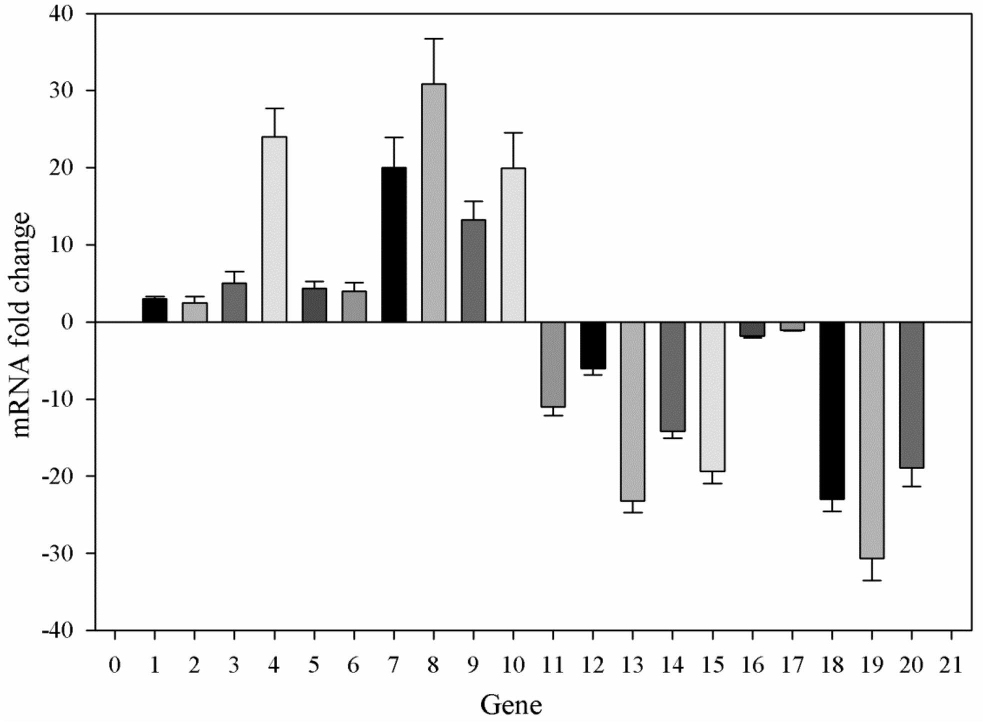

40
30
20
10
0
-10
-20
-30
-40
mRNA fold change
0
1
2
3
4
5
6
7
8
9
10
11
12
13
14
15
16
17
18
19
20
21
Gene
